# *Demuxafy*: improvement in droplet assignment by integrating multiple single-cell demultiplexing and doublet detection methods

**DOI:** 10.1186/s13059-024-03224-8

**Published:** 2024-04-15

**Authors:** Drew Neavin, Anne Senabouth, Himanshi Arora, Jimmy Tsz Hang Lee, Aida Ripoll-Cladellas, sc-eQTLGen Consortium, Lude Franke, Shyam Prabhakar, Chun Jimmie Ye, Davis J. McCarthy, Marta Melé, Martin Hemberg, Joseph E. Powell

**Affiliations:** 1 Garvan-Weizmann Centre for Cellular Genomics, Garvan Institute for Medical Research, Darlinghurst, NSW Australia; 2 Present address: Statewide Genomics at NSW Health Pathology, Sydney, NSW Australia; 3 Wellcome Sanger Institute, Wellcome Genome Campus, Hinxton, UK; 4 Life Sciences Department, Barcelona Supercomputing Center, Barcelona, Catalonia Spain; 5 Department of Genetics, University of Groningen, University Medical Center Groningen, Groningen, The Netherlands; 6 Spatial and Single Cell Systems Domain, Genome Institute of Singapore (GIS), Agency for Science, Technology and Research (A*STAR), Singapore, Republic of Singapore; 7 Population and Global Health, Lee Kong Chian School of Medicine, Nanyang Technological University, Singapore, Republic of Singapore; 8 Cancer Science Institute of Singapore, National University of Singapore, Singapore, Republic of Singapore; 9 Bakar Institute for Computational Health Sciences, University of California, San Francisco, CA USA; 10 Institute for Human Genetics, University of California, San Francisco, San Francisco, CA USA; 11 Division of Rheumatology, Department of Medicine, University of California, San Francisco, San Francisco, CA USA; 12 Chan Zuckerberg Biohub, San Francisco, CA USA; 13 Bioinformatics and Cellular Genomics, St Vincent’s Institute of Medical Research, Fitzroy, Australia; 14 Melbourne Integrative Genomics, School of BioSciences–School of Mathematics & Statistics, Faculty of Science, University of Melbourne, Melbourne, Australia; 15 Present address: The Gene Lay Institute of Immunology and Inflammation, Brigham and Women’s Hospital and Harvard Medical School, Boston, MA USA; 16 UNSW Cellular Genomics Futures Institute, University of New South Wales, Kensington, NSW Australia

**Keywords:** Single-cell analysis, Genetic demultiplexing, Doublet detecting

## Abstract

Recent innovations in single-cell RNA-sequencing (scRNA-seq) provide the technology to investigate biological questions at cellular resolution. Pooling cells from multiple individuals has become a common strategy, and droplets can subsequently be assigned to a specific individual by leveraging their inherent genetic differences. An implicit challenge with scRNA-seq is the occurrence of doublets—droplets containing two or more cells. We develop Demuxafy, a framework to enhance donor assignment and doublet removal through the consensus intersection of multiple demultiplexing and doublet detecting methods. Demuxafy significantly improves droplet assignment by separating singlets from doublets and classifying the correct individual.

**Supplementary Information:**

The online version contains supplementary material available at 10.1186/s13059-024-03224-8.

## Background

Droplet-based single-cell RNA sequencing (scRNA-seq) technologies have provided the tools to profile tens of thousands of single-cell transcriptomes simultaneously [1]. With these technological advances, combining cells from multiple samples in a single capture is common, increasing the sample size while simultaneously reducing batch effects, cost, and time. In addition, following cell capture and sequencing, the droplets can be demultiplexed—each droplet accurately assigned to each individual in the pool [2–7].

Many scRNA-seq experiments now capture upwards of 20,000 droplets, resulting in ~16% (3,200) doublets [8]. Current demultiplexing methods can also identify doublets—droplets containing two or more cells—from different individuals (heterogenic doublets). These doublets can significantly alter scientific conclusions if they are not effectively removed. Therefore, it is essential to remove doublets from droplet-based single-cell captures.

However, demultiplexing methods cannot identify droplets containing multiple cells from the same individual (homogenic doublets) and, therefore, cannot identify all doublets in a single capture. If left in the dataset, those doublets could appear as transitional cells between two distinct cell types or a completely new cell type. Accordingly, additional methods have been developed to identify heterotypic doublets (droplets that contain two cells from different cell types) by comparing the transcriptional profile of each droplet to doublets simulated from the dataset [9–15]. It is important to recognise that demultiplexing methods achieve two functions—segregation of cells from different donors and separation of singlets from doublets—while doublet detecting methods solely classify singlets versus doublets.

Therefore, demultiplexing and transcription-based doublet detecting methods provide complementary information to improve doublet detection, providing a cleaner dataset and more robust scientific results. There are currently five genetic-based demultiplexing [2–7, 16] and seven transcription-based doublet-detecting methods implemented in various languages [9–15]. Under different scenarios, each method is subject to varying performance and, in some instances, biases in their ability to accurately assign cells or detect doublets from certain conditions. The best combination of methods is currently unclear but will undoubtedly depend on the dataset and research question.

**Fig. 1:**
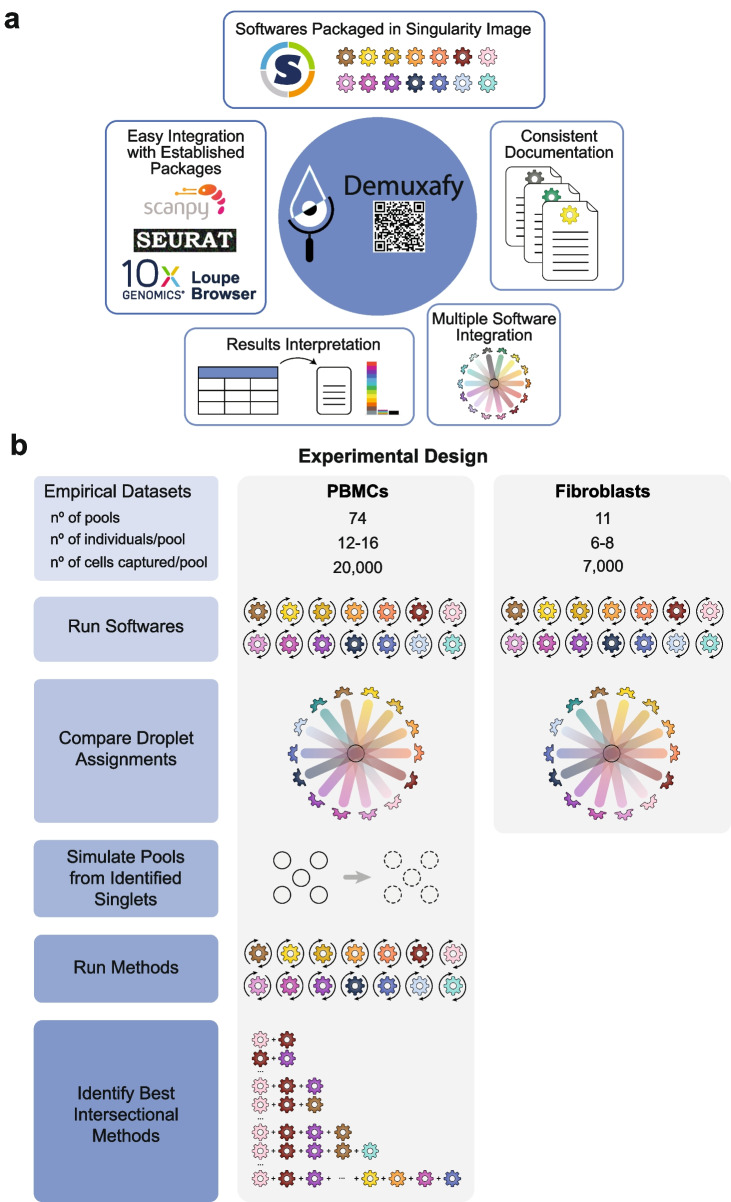
Study design and qualitative method classifications. **a** Demuxafy is a platform to perform demultiplexing and doublet detecting with consistent documentation. Demuxafy also provides wrapper scripts to quickly summarize the results from each method and assign clusters to each individual with reference genotypes when a reference-free demultiplexing method is used. Finally, Demuxafy provides a script to easily combine the results from multiple different methods into a single data frame and it provides a final assignment for each droplet based on the combination of multiple methods. In addition, Demuxafy provides summaries of the number of droplets classified as singlets or doublets by each method and a summary of the number of droplets assigned to each individual by each of the demultiplexing methods. **b** Two datasets are included in this analysis - a PBMC dataset and a fibroblast dataset. The PBMC dataset contains 74 pools that captured approximately 20,000 droplets each with 12-16 donor cells multiplexed per pool. The fibroblast dataset contains 11 pools of roughly 7,000 droplets per pool with sizes ranging from six to eight donors per pool. All pools were processed by all demultiplexing and doublet detecting methods and the droplet and donor classifications were compared between the methods and between the PBMCs and fibroblasts. Then the PBMC droplets that were classified as singlets by all methods were taken as ‘true singlets’ and used to generate new pools in silico. Those pools were then processed by each of the demultiplexing and doublet detecting methods and intersectional combinations of demultiplexing and doublet detecting methods were tested for different experimental designs

Therefore, we set out to identify the best combination of genetic-based demultiplexing and transcription-based doublet-detecting methods to remove doublets and partition singlets from different donors correctly. In addition, we have developed a software platform (*Demuxafy*) that performs these intersectional methods and provides additional commands to simplify the execution and interpretation of results for each method (Fig. 1a).

To compare the demultiplexing and doublet detecting methods, we utilised two large, multiplexed datasets—one that contained ~1.4 million peripheral blood mononuclear cells (PBMCs) from 1,034 donors [17] and one with ~94,000 fibroblasts from 81 donors [18]. We used the true singlets from the PBMC dataset to generate new in silico pools to assess the performance of each method and the multi-method intersectional combinations (Fig. 1b).

Here, we compare 14 demultiplexing and doublet detecting methods with different methodological approaches, capabilities, and intersectional combinations. Seven of those are demultiplexing methods (*Demuxalot* [6], *Demuxlet* [3], *Dropulation* [5], *Freemuxlet* [16], *ScSplit* [7], *Souporcell* [4], and *Vireo* [2]) which leverage the common genetic variation between individuals to identify cells that came from each individual and to identify heterogenic doublets. The seven remaining methods (*DoubletDecon* [9], *DoubletDetection* [14], *DoubletFinder* [10], *ScDblFinder* [11], *Scds* [12], *Scrublet* [13], and *Solo* [15]) identify doublets based on their similarity to simulated doublets generated by adding the transcriptional profiles of two randomly selected droplets in the dataset. These methods assume that the proportion of real doublets in the dataset is low, so combining any two droplets will likely represent the combination of two singlets.

We identify critical differences in the performance of demultiplexing and doublet detecting methods to classify droplets correctly. In the case of the demultiplexing techniques, their performance depends on their ability to identify singlets from doublets and assign a singlet to the correct individual. For doublet detecting methods, the performance is based solely on their ability to differentiate a singlet from a doublet. We identify limitations in identifying specific doublet types and cell types by some methods. In addition, we compare the intersectional combinations of these methods for multiple experimental designs and demonstrate that intersectional approaches significantly outperform all individual techniques. Thus, the intersectional methods provide enhanced singlet classification and doublet removal—a critical but often under-valued step of droplet-based scRNA-seq processing. Our results demonstrate that intersectional combinations of demultiplexing and doublet detecting software provide significant advantages in droplet-based scRNA-seq preprocessing that can alter results and conclusions drawn from the data. Finally, to provide easy implementation of our intersectional approach, we provide *Demuxafy* (https://demultiplexing-doublet-detecting-docs.readthedocs.io/en/latest/index.html) a complete platform to perform demultiplexing and doublet detecting intersectional methods (Fig. 1a).

## Results

### Study design

To evaluate demultiplexing and doublet detecting methods, we developed an experimental design that applies the different techniques to empirical pools and pools generated in silico from the combination of true singlets—droplets identified as singlets by every method (Fig. 1a). For the first phase of this study, we used two empirical multiplexed datasets—the peripheral blood mononuclear cell (PBMC) dataset containing ~1.4 million cells from 1034 donors and a fibroblast dataset of ~94,000 cells from 81 individuals (Additional file 1: Table S1). We chose these two cell systems to assess the methods in heterogeneous (PBMC) and homogeneous (fibroblast) cell types.

### Demultiplexing and doublet detecting methods perform similarly for heterogeneous and homogeneous cell types

**Fig. 2:**
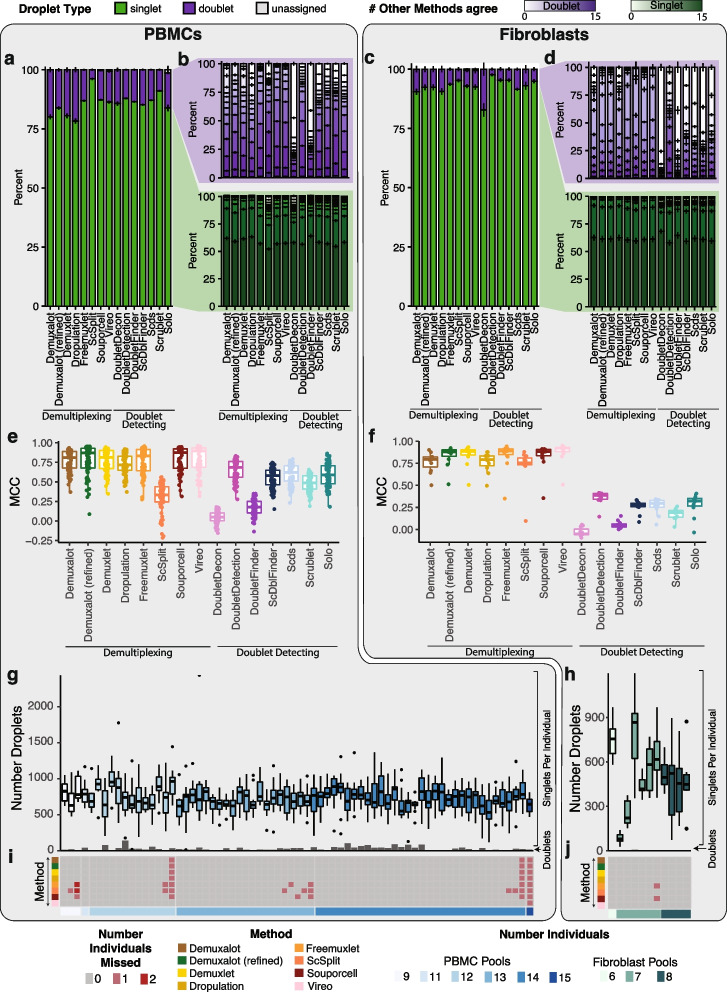
Demultiplexing and Doublet Detecting Method Performance Comparison. **a** The proportion of droplets classified as singlets and doublets by each method in the PBMCs. **b** The number of other methods that classified the singlets and doublets identified by each method in the PBMCs. **c** The proportion of droplets classified as singlets and doublets by each method in the fibroblasts. **d** The number of other methods that classified the singlets and doublets identified by each method in the fibroblasts. **e**-**f** The performance of each method when the majority classification of each droplet is considered the correct annotation in the PBMCs (**e**) and fibroblasts (**f**). **g**-**h** The number of droplets classified as singlets (box plots) and doublets (bar plots) by all methods in the PBMC (**g**) and fibroblast (**h**) pools. **i**-**j** The number of donors that were not identified by each method in each pool for PBMCs (**i**) and fibroblasts (**j**). PBMC: peripheral blood mononuclear cell. MCC: Matthew’s correlationcoefficient

We applied the demultiplexing methods (*Demuxalot*, *Demuxlet*, *Dropulation*, *Freemuxlet*, *ScSplit*, *Souporcell*, and *Vireo*) and doublet detecting methods (*DoubletDecon*, *DoubletDetection*, *DoubletFinder*, *ScDblFinder*, *Scds*, *Scrublet*, and *Solo*) to the two datasets and assessed the results from each method. We first compared the droplet assignments by identifying the number of singlets and doublets identified by a given method that were consistently annotated by all methods (Fig. 2a–d). We also identified the percentage of droplets that were annotated consistently between pairs of methods (Additional file 2: Fig S1). In the cases where two demultiplexing methods were compared to one another, both the droplet type (singlet or doublet) and the assignment of the droplet to an individual had to match to be considered in agreement. In all other comparisons (i.e. demultiplexing versus doublet detecting and doublet detecting versus doublet detecting), only the droplet type (singlet or doublet) was considered for agreement since doublet detecting methods cannot annotate donor assignment. We found that the two method types were more similar to other methods of the same type (i.e., demultiplexing versus demultiplexing and doublet detecting versus doublet detecting) than they were to methods from a different type (demultiplexing methods versus doublet detecting methods; Supplementary Fig 1). We found that the similarity of the demultiplexing and doublet detecting methods was consistent in the PBMC and fibroblast datasets (Pearson correlation R = 0.78, *P*-value < 2×10^−16^; Fig S1a-c). In addition, demultiplexing methods were more similar than doublet detecting methods for both the PBMC and fibroblast datasets (Wilcoxon rank-sum test: *P* < 0.01; Fig. 2a–b and Additional file 2: Fig S1).

The number of unique molecular identifiers (UMIs) and genes decreased in droplets that were classified as singlets by a larger number of methods while the mitochondrial percentage increased in both PBMCs and fibroblasts (Additional file 2: Fig S2).

We next interrogated the performance of each method using the Matthew’s correlation coefficient (MCC) to calculate the consistency between *Demuxify* and true droplet classification. We identified consistent trends in the MCC scores for each method between the PBMCs (Fig. 2e) and fibroblasts (Fig. 2f). These data indicate that the methods behave similarly, relative to one another, for heterogeneous and homogeneous datasets.

Next, we sought to identify the droplets concordantly classified by all demultiplexing and doublet detecting methods in the PBMC and fibroblast datasets. On average, 732 singlets were identified for each individual by all the methods in the PBMC dataset. Likewise, 494 droplets were identified as singlets for each individual by all the methods in the fibroblast pools. However, the concordance of doublets identified by all methods was very low for both datasets (Fig. 2e–f). Notably, the consistency of classifying a droplet as a doublet by all methods was relatively low (Fig. 2b,d,g, and h). This suggests that doublet identification is not consistent between all the methods. Therefore, further investigation is required to identify the reasons for these inconsistencies between methods. It also suggests that combining multiple methods for doublet classification may be necessary for more complete doublet removal. Further, some methods could not identify all the individuals in each pool (Fig. 2i–j). The non-concordance between different methods demonstrates the need to effectively test each method on a dataset where the droplet types are known.

### Computational resources vary for demultiplexing and doublet detecting methods

We recorded each method’s computational resources for the PBMC pools, with ~20,000 cells captured per pool (Additional file 1: Table S1). Of the demultiplexing methods, *ScSplit* took the most time (multiple days) and required the most steps, but *Demuxalot*, *Demuxlet*, and *Freemuxlet* used the most memory. *Solo* took the longest time (median 13 h) and most memory to run for the doublet detecting methods but is the only method built to be run directly from the command line, making it easy to implement (Additional file 2: Fig S3).

### Generate pools with known singlets and doublets

However, there is no gold standard to identify which droplets are singlets or doublets. Therefore, in the second phase of our experimental design (Fig. 1b), we used the PBMC droplets classified as singlets by all methods to generate new pools in silico. We chose to use the PBMC dataset since our first analyses indicated that method performance is similar for homogeneous (fibroblast) and heterogeneous (PBMC) cell types (Fig. 2 and Additional file 2: Fig S1) and because we had many more individuals available to generate in silico pools from the PBMC dataset (Additional file 1: Table S1).

**Fig. 3:**
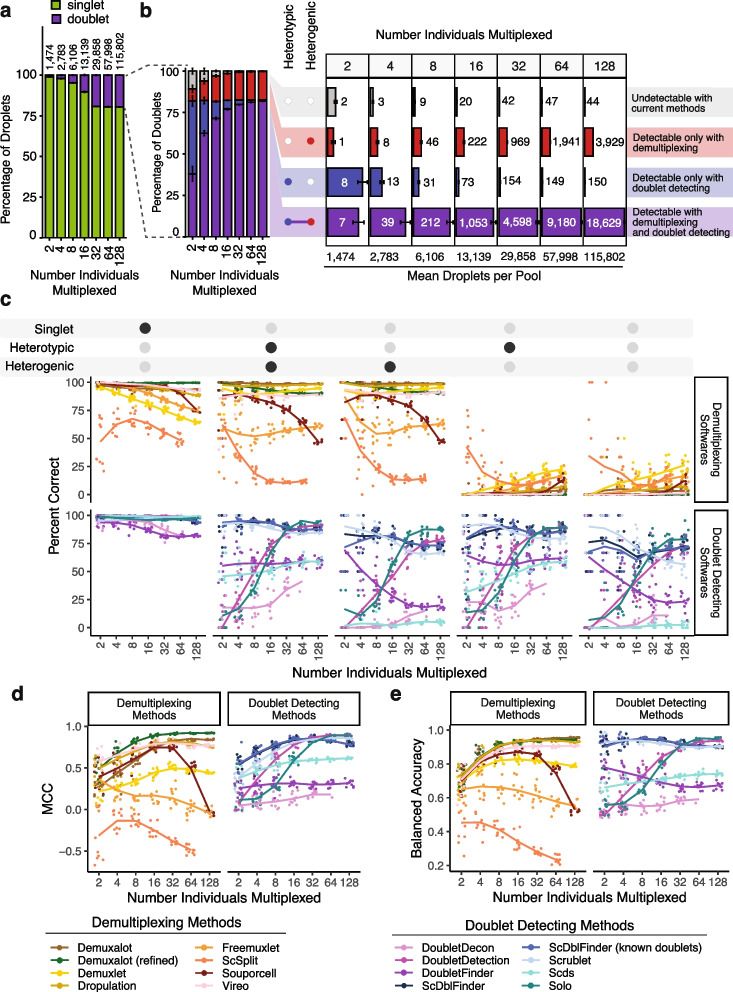
In silico Pool Doublet Annotation and Method Performance. **a** The percent of singlets and doublets in the in -silico pools - separated by the number of multiplexed individuals per pool. **b** The percentage and number of doublets that are heterogenic (detectable by demultiplexing methods), heterotypic (detectable by doublet detecting methods), both (detectable by either method category) and neither (not detectable with current methods) for each multiplexed pool size. **c** Percent of droplets that each of the demultiplexing and doublet detecting methods classified correctly for singlets and doublet subtypes for different multiplexed pool sizes. **d** Matthew’s Correlation Coefficient (MCC) for each of the methods for each of the multiplexed pool sizes. **e** Balanced accuracy for each of the methods for each of the multiplexed pool sizes

We generated 70 pools—10 each of pools that included 2, 4, 8, 16, 32, 64, or 128 individuals (Additional file 1: Table S2). We assume a maximum 20% doublet rate as it is unlikely researchers would use a technology that has a higher doublet rate (Fig. 3a).

We used azimuth to classify the PBMC cell types for each droplet used to generate the in silico pools [19] (Additional file 2: Fig S4). As these pools have been generated in silico using empirical singlets that have been well annotated, we next identified the proportion of doublets in each pool that were heterogenic, heterotypic, both, and neither. This approach demonstrates that a significant percentage of doublets are only detectable by doublet detecting methods (homogenic and heterotypic) for pools with 16 or fewer donors multiplexed (Fig. 3b).

While the total number of doublets that would be missed if only using demultiplexing methods appears small for fewer multiplexed individuals (Fig. 3b), it is important to recognise that this is partly a function of the ~732 singlet cells per individual used to generate these pools. Hence, the in silico pools with fewer individuals also have fewer cells. Therefore, to obtain numbers of doublets that are directly comparable to one another, we calculated the number of each doublet type that would be expected to be captured with 20,000 cells when 2, 4, 8, 16, or 32 individuals were multiplexed (Additional file 2: Fig S5). These results demonstrate that many doublets would be falsely classified as singlets since they are homogenic when just using demultiplexing methods for a pool of 20,000 cells captured with a 16% doublet rate (Additional file 2: Fig S5). However, as more individuals are multiplexed, the number of droplets that would not be detectable by demultiplexing methods (homogenic) decreases. This suggests that typical workflows that use only one demultiplexing method to remove doublets from pools that capture 20,000 droplets with 16 or fewer multiplexed individuals fail to adequately remove between 173 (16 multiplexed individuals) and 1,325 (2 multiplexed individuals) doublets that are homogenic and heterotypic which could be detected by doublet detecting methods (Additional file 2: Fig S5). Therefore, a technique that uses both demultiplexing and doublet detecting methods in parallel will complement more complete doublet removal methods. Consequently, we next set out to identify the demultiplexing and doublet detecting methods that perform the best on their own and in concert with other methods.

### Doublet and singlet droplet classification effectiveness varies for demultiplexing and doublet detecting methods

#### Demultiplexing methods fail to classify homogenic doublets

We next investigated the percentage of the droplets that were correctly classified by each demultiplexing and doublet detecting method. In addition to the seven demultiplexing methods, we also included *Demuxalot* with the additional steps to refine the genotypes that can then be used for demultiplexing—*Demuxalot* (refined). Demultiplexing methods correctly classify a large portion of the singlets and heterogenic doublets (Fig. 3c). This pattern is highly consistent across different cell types, with the notable exceptions being decreased correct classifications for erythrocytes and platelets when greater than 16 individuals are multiplexed (Additional file 2: Fig S6).

However, *Demuxalot* consistently demonstrates the highest correct heterogenic doublet classification. Further, the percentage of the heterogenic doublets classified correctly by *Souporcell* decreases when large numbers of donors are multiplexed. *ScSplit* is not as effective as the other demultiplexing methods at classifying heterogenic doublets, partly due to the unique doublet classification method, which assumes that the doublets will generate a single cluster separate from the donors (Table 1). Importantly, the demultiplexing methods identify almost none of the homogenic doublets for any multiplexed pool size—demonstrating the need to include doublet detecting methods to supplement the demultiplexing method doublet detection.

**Table 1:**
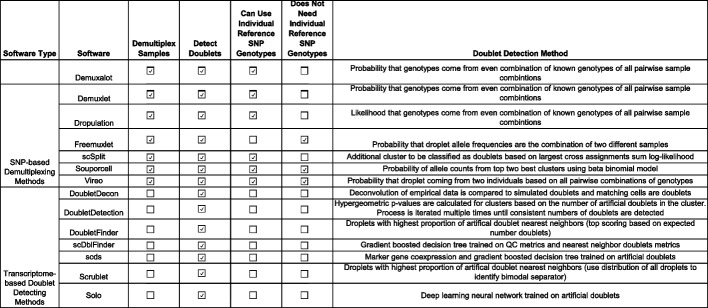
Demultiplexing and doublet detecting method characteristics, requirements and doublet detection methodologies

#### Doublet detecting method classification performances vary greatly

In addition to assessing each of the methods with default settings, we also evaluated *ScDblFinder* with ‘known doublets’ provided. This method can take already known doublets and use them when detecting doublets. For these cases, we used the droplets that were classified as doublets by all the demultiplexing methods as ‘known doublets’.

Most of the methods classified a similarly high percentage of singlets correctly, with the exceptions of *DoubletDecon* and *DoubletFinder* for all pool sizes (Fig. 3c). However, unlike the demultiplexing methods, there are explicit cell-type-specific biases for many of the doublet detecting methods (Additional file 2: Fig S7). These differences are most notable for cell types with fewer cells (i.e. ASDC and cDC2) and proliferating cells (i.e. CD4 Proliferating, CD8 Proliferating, and NK Proliferating). Further, all of the softwares demonstrate high correct percentages for some cell types including CD4 Naïve and CD8 Naïve (Additional file 2: Fig S7).

As expected, all doublet detecting methods identified heterotypic doublets more effectively than homotypic doublets (Fig. 3c). However, *ScDblFinder *and* Scrublet* classified the most doublets correctly across all doublet types for pools containing 16 individuals or fewer. *Solo* was more effective at identifying doublets than *Scds* for pools containing more than 16 individuals. It is also important to note that it was not feasible to run *DoubletDecon* for the largest pools containing 128 multiplexed individuals and an average of 115,802 droplets (range: 113,594–119,126 droplets). *ScDblFinder* performed similarly when executed with and without known doublets (Pearson correlation *P* = 2.5 × 10^-40^). This suggests that providing known doublets to *ScDblFinder* does not offer an added benefit.

#### Performances vary between demultiplexing and doublet detecting method and across the number of multiplexed individuals

We assessed the overall performance of each method with two metrics: the balanced accuracy and the MCC. We chose to use balanced accuracy since, with unbalanced group sizes, it is a better measure of performance than accuracy itself. Further, the MCC has been demonstrated as a more reliable statistical measure of performance since it considers all possible categories—true singlets (true positives), false singlets (false positives), true doublets (true negatives), and false doublets (false negatives). Therefore, a high score on the MCC scale indicates high performance in each metric. However, we provide additional performance metrics for each method (Additional file 1: Table S3). For demultiplexing methods, both the droplet type (singlet or doublet) and the individual assignment were required to be considered a ‘true singlet’. In contrast, only the droplet type (singlet or doublet) was needed for doublet detection methods.

The MCC and balanced accuracy metrics are similar (Spearman’s *⍴* = 0.87; *P* < 2.2 × 10^-308^). Further, the performance of *Souporcell* decreases for pools with more than 32 individuals multiplexed for both metrics (Student’s *t*-test for MCC: *P* < 1.1 × 10^-9^ and balanced accuracy: *P* < 8.1 × 10^-11^). *Scds*, *ScDblFinder*, and *Scrublet* are among the top-performing doublet detecting methods Fig. 3d–e).

Overall, between 0.4 and 78.8% of droplets were incorrectly classified by the demultiplexing or doublet detecting methods depending on the technique and the multiplexed pool size (Additional file 2: Fig S8). *Demuxalot *(refined) and *DoubletDetection* demonstrated the lowest percentage of incorrect droplets with about 1% wrong in the smaller pools (two multiplexed individuals) and about 3% incorrect in pools with at least 16 multiplexed individuals. Since some transitional states and cell types are present in low percentages in total cell populations (i.e. ASDCs at 0.02%), incorrect classification of droplets could alter scientific interpretations of the data, and it is, therefore, ideal for decreasing the number of erroneous assignments as much as possible.

#### False singlets and doublets demonstrate different metrics than correctly classified droplets

We next asked whether specific cell metrics might contribute to false singlet and doublet classifications for different methods. Therefore, we compared the number of genes, number of UMIs, mitochondrial percentage and ribosomal percentage of the false singlets and doublets to equal numbers of correctly classified cells for each demultiplexing and doublet detecting method.

The number of UMIs (Additional file 2: Fig S9 and Additional file 1: Table S4) and genes (Additional file 2: Fig S10 and Additional file 1: Table S5) demonstrated very similar distributions for all comparisons and all methods (Spearman *⍴* = 0.99, *P* < 2.2 × 10^-308^). The number of UMIs and genes were consistently higher in false singlets and lower in false doublets for most demultiplexing methods except some smaller pool sizes (Additional file 2: Fig S9a and Additional file 2: Fig S10a; Additional file 1: Table S4 and Additional file 1: Table S5). The number of UMIs and genes was consistently higher in droplets falsely classified as singlets by the doublet detecting methods than the correctly identified droplets (Additional file 2: Fig S9b and Additional file 2: Fig S10b; Additional file 1: Table S4 and Additional file 1: Table S5). However, there was less consistency in the number of UMIs and genes detected in false singlets than correctly classified droplets between the different doublet detecting methods (Additional file 2: Fig S9b and Additional file 2: Fig S10b; Additional file 1: Table S4 and Additional file 1: Table S5).

The ribosomal percentage of the droplets falsely classified as singlets or doublets is similar to the correctly classified droplets for most methods—although they are statistically different for larger pool sizes (Additional file 2: Fig S11a and Additional file 1: Table S6). However, the false doublets classified by some demultiplexing methods (*Demuxalot*, *Demuxalot* (refined), *Demuxlet*, *ScSplit*, *Souporcell*, and *Vireo*) demonstrated higher ribosomal percentages. Some doublet detecting methods (*ScDblFinder*, *ScDblFinder* with known doublets and *Solo)* demonstrated higher ribosomal percentages for the false doublets while other demonstrated lower ribosomal percentages (*DoubletDecon*, *DoubletDetection*, and *DoubletFinder*; Additional file 2: Fig S11b and Additional file 1: Table S6).

Like the ribosomal percentage, the mitochondrial percentage in false singlets is also relatively similar to correctly classified droplets for both demultiplexing (Additional file 2: Fig S12a and Additional file 1: Table S7) and doublet detecting methods (Additional file 2: Fig S12b). The mitochondrial percentage for false doublets is statistically lower than the correctly classified droplets for a few larger pools for *Freemuxlet*, *ScSplit*, and *Souporcell*. The doublet detecting method *Solo* also demonstrates a small but significant decrease in mitochondrial percentage in the false doublets compared to the correctly annotated droplets. However, other doublet detecting methods including *DoubletFinder* and the larger pools of most other methods demonstrated a significant increase in mitochondrial percent in the false doublets compared to the correctly annotated droplets (Additional file 2: Fig S12b).

Overall, these results demonstrate a strong relationship between the number of genes and UMIs and limited influence of ribosomal or mitochondrial percentage in a droplet and false classification, suggesting that the number of genes and UMIs can significantly bias singlet and doublet classification by demultiplexing and doublet detecting methods.

#### Ambient RNA, number of reads per cell, and uneven pooling impact method performance

To further quantify the variables that impact the performance of each method, we simulated four conditions that could occur with single-cell RNA-seq experiments: (1) decreased number of reads (reduced 50%), (2) increased ambient RNA (10%, 20% and 50%), (3) increased mitochondrial RNA (5%, 10% and 25%) and 4) uneven donor pooling from single donor spiking (0.5 or 0.75 proportion of pool from one donor). We chose these scenarios because they are common technical effects that can occur.

We observed a consistent decrease in the demultiplexing method performance when the number of reads were decreased by 50% but the degree of the effect varied for each method and was larger in pools containing more multiplexed donors (Additional file 2: Fig S13a and Additional file 1: Table S8). Decreasing the number of reads did not have a detectable impact on the performance of the doublet detecting methods.

Simulating additional ambient RNA (10%, 20%, or 50%) decreased the performance of all the demultiplexing methods (Additional file 2: Fig S13b and Additional file 1: Table S9) but some were unimpacted in pools that had 16 or fewer individuals multiplexed (*Souporcell* and *Vireo*). The performance of some of the doublet detecting methods were impacted by the ambient RNA but the performance of most methods did not decrease. *Scrublet* and *ScDblFinder* were the doublet detecting methods most impacted by ambient RNA but only in pools with at least 32 multiplexed donors (Additional file 2: Fig S13b and Additional file 1: Table S9).

Increased mitochondrial percent did not impact the performance of demultiplexing or doublet detecting methods (Additional file 2: Fig S13c and Additional file 1: Table S10).

We also tested whether experimental designs that pooling uneven proportions of donors would alter performance. We tested scenarios where either half the pool was composed of a single donor (0.5 spiked donor proportion) or where three quarters of the pool was composed of a single donor. This experimental design significantly reduced the demultiplexing method performance (Additional file 2: Fig S13d and Additional file 1: Table S11) with the smallest influence on *Freemuxlet*. The performance of most of the doublet detecting methods were unimpacted except for *DoubletDetection* that demonstrated significant decreases in performance in pools where at least 16 donors were multiplexed. Intriguingly, the performance of *Solo* increased with the spiked donor pools when the pools consisted of 16 donors or less.

**Fig. 4:**
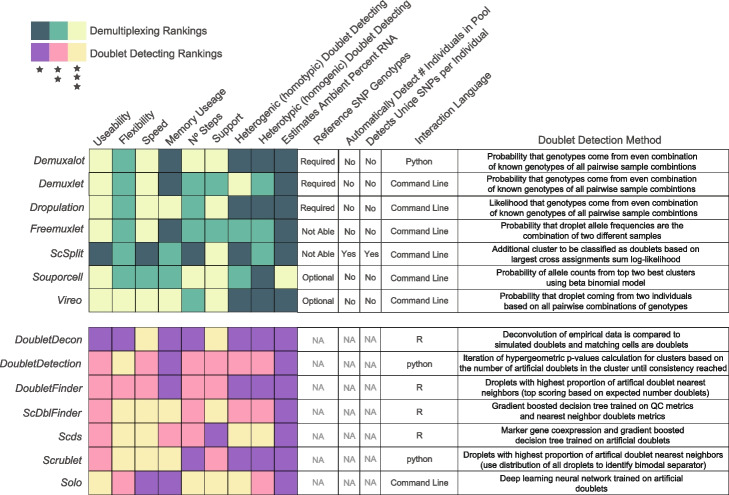
Assessment of each of the demultiplexing and doublet detecting methods. Assessments of a variety of metrics for each of the demultiplexing (top) and doublet detecting (bottom) methods

Our results demonstrate significant differences in overall performance between different demultiplexing and doublet detecting methods. We further noticed some differences in the use of the methods. Therefore, we have accumulated these results and each method’s unique characteristics and benefits in a heatmap for visual interpretation (Fig. 4).

### Framework for improving singlet classifications via method combinations

After identifying the demultiplexing and doublet detecting methods that performed well individually, we next sought to test whether using intersectional combinations of multiple methods would enhance droplet classifications and provide a software platform—*Demuxafy*—capable of supporting the execution of these intersectional combinations.

We recognise that different experimental designs will be required for each project. As such, we considered this when testing combinations of methods. We considered multiple experiment designs and two different intersectional methods: (1) more than half had to classify a droplet as a singlet to be called a singlet and (2) at least half of the methods had to classify a droplet as a singlet to be called a singlet. Significantly, these two intersectional methods only differ when an even number of methods are being considered. For combinations that include demultiplexing methods, the individual called by the majority of the methods is the individual used for that droplet. When ties occur, the individual is considered ‘unassigned’.

#### Combining multiple doublet detecting methods improve doublet removal for non-multiplexed experimental designs

**Fig. 5:**
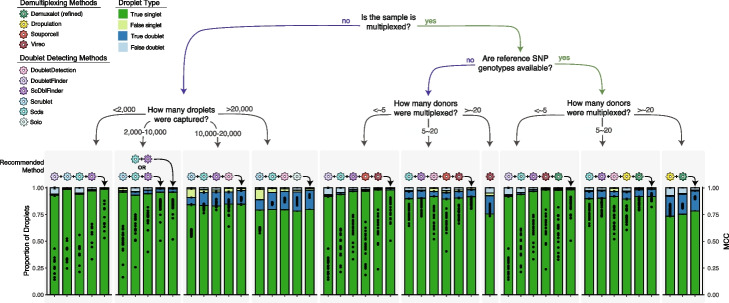
Recommended Method Combinations Dependent on Experimental Design. Method combinations are provided for different experimental designs, including those that are not multiplexed (left) and multiplexed (right), including experiments that have reference SNP genotypes available vs those that do not and finally, multiplexed experiments with different numbers of individuals multiplexed. The each bar represents either a single method (shown with the coloured icon above the bar) or a combination of methods (shown with the addition of the methods and an arrow indicating the bar). The proportion of true singlets, true doublets, false singlets and false doublets for each method or combination of methods is shown with the filled barplot and the MCC is shown with the black points overlaid on the barplot. MCC: Matthew’s Correlation Coefficient

For the non-multiplexed experimental design, we considered all possible method combinations (Additional file 1: Table S12). We identified important differences depending on the number of droplets captured and have provided recommendations accordingly. We identified that *DoubletFinder*, *Scrublet*, *ScDblFinder* and *Scds* is the ideal combination for balanced droplet calling when less than 2,000 droplets are captured. *Scds* and *ScDblFinder* or *Scrublet*, *Scds* and *ScDblFinder* is the best combination when 2,000–10,000 droplets are captured. *Scds*, *Scrublet, ScDblFinder* and *DoubletDetection* is the best combination when 10,000–20,000 droplets are captured and *Scrublet*, *Scds*, *DoubletDetection* and *ScDblFinder*. It is important to note that even a slight increase in the MCC significantly impacts the number of true singlets and true doublets classified with the degree of benefit highly dependent on the original method performance. The combined method increases the MCC compared to individual doublet detecting methods on average by 0.11 and up to 0.33—a significant improvement in the MCC (*t*-test FDR < 0.05 for 95% of comparisons). For all combinations, the intersectional droplet method requires more than half of the methods to consider the droplet a singlet to classify it as a singlet (Fig. 5).

#### Demuxafy performs better than Chord

*Chord* is an ensemble machine learning doublet detecting method that uses *Scds* and *DoubletFinder* to identify doublets. We compared *Demuxafy* using *Scds* and *DoubletFinder* to *Chord* and identified that *Demuxafy* outperformed Chord in pools that contained at least eight donors and was equivalent in pools that contained less than eight donors (Additional file 2: Fig S14). This is because Chord classifies more droplets as false singlets and false doublets than *Demuxafy*. In addition, *Chord* failed to complete for two of the pools that contained 128 multiplexed donors.

#### Combining multiple demultiplexing and doublet detecting methods improve doublet removal for multiplexed experimental designs

For experiments where 16 or fewer individuals are multiplexed with reference SNP genotypes available, we considered all possible combinations between the demultiplexing and doublet detecting methods except *ScDblFinder* with known doublets due to its highly similar performance to *ScDblFinder* (Fig 3; Additional file 1: Table S13). The best combinations are *DoubletFinder*, *Scds*, *ScDblFinder*, *Vireo* and *Demuxalot* (refined) (<~5 donors) and *Scrublet*, *ScDblFinder*, *DoubletDetection*, *Dropulation* and *Demuxalot* (refined) (Fig. 5). These intersectional methods increase the MCC compared to the individual methods (*t*-test FDR < 0.05), generally resulting in increased true singlets and doublets compared to the individual methods. The improvement in MCC depends on every single method’s performance but, on average, increases by 0.22 and up to 0.71. For experiments where the reference SNP genotypes are unknown, the individuals multiplexed in the pool with 16 or fewer individuals multiplexed, *DoubletFinder*, *ScDblFinder, Souporcell* and *Vireo* (<~5 donors) and *Scds*, *ScDblFinder*, *DoubletDetection*, *Souporcell* and *Vireo* are the ideal methods (Fig. 5). These intersectional methods again significantly increase the MCC up to 0.87 compared to any of the individual techniques that could be used for this experimental design (*t*-test FDR < 0.05 for 94.2% of comparisons). In both cases, singlets should only be called if more than half of the methods in the combination classify the droplet as a singlet.

#### Combining multiple demultiplexing methods improves doublet removal for large multiplexed experimental designs

For experiments that multiplex more than 16 individuals, we considered the combinations between all demultiplexing methods (Additional file 1: Table S14) since only a small proportion of the doublets would be undetectable by demultiplexing methods (droplets that are homogenic; Fig 3b). To balance doublet removal and maintain true singlets, we recommend the combination of *Demuxalot* (refined) and *Dropulation*. These method combinations significantly increase the MCC by, on average, 0.09 compared to all the individual methods (*t*-test FDR < 0.05). This substantially increases true singlets and true doublets relative to the individual methods. If reference SNP genotypes are not available for the individuals multiplexed in the pools, *Vireo* performs the best (≥ 16 multiplexed individuals; Fig. 5). This is the only scenario in which executing a single method is advantageous to a combination of methods. This is likely due to the fact that most of the methods perform poorly for larger pool sizes (Fig. 3c).

These results collectively demonstrate that, regardless of the experimental design, demultiplexing and doublet detecting approaches that intersect multiple methods significantly enhance droplet classification. This is consistent across different pool sizes and will improve singlet annotation.

### Demuxafy improves doublet removal and improves usability

To make our intersectional approaches accessible to other researchers, we have developed *Demuxafy *(https://demultiplexing-doublet-detecting-docs.readthedocs.io/en/latest/index.html) - an easy-to-use software platform powered by Singularity. This platform provides the requirements and instructions to execute each demultiplexing and doublet detecting methods. In addition, *Demuxafy* provides wrapper scripts that simplify method execution and effectively summarise results. We also offer tools that help estimate expected numbers of doublets and provide method combination recommendations based on scRNA-seq pool characteristics. *Demuxafy* also combines the results from multiple different methods, provides classification combination summaries, and provides final integrated combination classifications based on the intersectional techniques selected by the user. The significant advantages of *Demuxafy* include a centralised location to execute each of these methods, simplified ways to combine methods with an intersectional approach, and summary tables and figures that enable practical interpretation of multiplexed datasets (Fig. 1a).

## Discussion

Demultiplexing and doublet detecting methods have made large-scale scRNA-seq experiments achievable. However, many demultiplexing and doublet detecting methods have been developed in the recent past, and it is unclear how their performances compare. Further, the demultiplexing techniques best detect heterogenic doublets while doublet detecting methods identify heterotypic doublets. Therefore, we hypothesised that demultiplexing and doublet detecting methods would be complementary and be more effective at removing doublets than demultiplexing methods alone.

Indeed, we demonstrated the benefit of utilising a combination of demultiplexing and doublet detecting methods. The optimal intersectional combination of methods depends on the experimental design and capture characteristics. Our results suggest super loaded captures—where a high percentage of doublets is expected—will benefit from multiplexing. Further, when many donors are multiplexed (>16), doublet detecting is not required as there are few doublets that are homogenic and heterotypic.

We have﻿ provided different method combination recommendations based on the experimental design. This decision is highly dependent on the research question.

## Conclusions

Overall, our results provide researchers with important demultiplexing and doublet detecting performance assessments and combinatorial recommendations. Our software platform, *Demuxafy* (https://demultiplexing-doublet-detecting-docs.readthedocs.io/en/latest/index.html), provides a simple implementation of our methods in any research lab around the world, providing cleaner scRNA-seq datasets and enhancing interpretation of results.

## Methods

### Data

#### PBMC scRNA-seq data

Blood samples were collected and processed as described previously [17]. Briefly, mononuclear cells were isolated from whole blood samples and stored in liquid nitrogen until thawed for scRNA-seq capture. Equal numbers of cells from 12 to 16 samples were multiplexed per pool and single-cell suspensions were super loaded on a Chromium Single Cell Chip A (10x Genomics) to capture 20,000 droplets per pool. Single-cell libraries were processed per manufacturer instructions and the 10× Genomics Cell Ranger Single Cell Software Suite (v 2.2.0) was used to process the data and map it to GRCh38. Cellbender v0.1.0 was used to identify empty droplets. Almost all droplets reported by Cell Ranger were identified to contain cells by Cellbender (mean: 99.97%). The quality control metrics of each pool are demonstrated in Additional file 2: Fig S15.

#### PBMC DNA SNP genotyping

SNP genotype data were prepared as described previously [17]. Briefly, DNA was extracted from blood with the QIAamp Blood Mini kit and genotyped on the Illumina Infinium Global Screening Array. SNP genotypes were processed with Plink and GCTA before imputing on the Michigan Imputation Server using Eagle v2.3 for phasing and Minimac3 for imputation based on the Haplotype Reference Consortium panel (HRCr1.1). SNP genotypes were then lifted to hg38 and filtered for > 1% minor allele frequency (MAF) and an *R*^2^ > 0.3.

#### Fibroblast scRNA-seq data

The fibroblast scRNA-seq data has been described previously [18]. Briefly, human skin punch biopsies from donors over the age of 18 were cultured in DMEM high glucose supplemented with 10% fetal bovine serum (FBS), L-glutamine, 100 U/mL penicillin and 100 μg/mL (Thermo Fisher Scientific, USA).

For scRNA-seq, viable cells were flow sorted and single cell suspensions were loaded onto a 10× Genomics Single Cell 3’ Chip and were processed per 10× instructions and the Cell Ranger Single Cell Software Suite from 10× Genomics was used to process the sequencing data into transcript count tables as previously described [18]. Cellbender v0.1.0 was used to identify empty droplets. Almost all droplets reported by Cell Ranger were identified to contain cells by Cellbender (mean: 99.65%). The quality control metrics of each pool are demonstrated in Additional file 2: Fig S16.

#### Fibroblast DNA SNP genotyping

The DNA SNP genotyping for fibroblast samples has been described previously [18]. Briefly, DNA from each donor was genotyped on an Infinium HumanCore-24 v1.1 BeadChip (Illumina). GenomeStudioTM V2.0 (Illumina), Plink and GenomeStudio were used to process the SNP genotypes. Eagle V2.3.5 was used to phase the SNPs and it was imputed with the Michigan Imputation server using minimac3 and the 1000 genome phase 3 reference panel as described previously [18].

### Demultiplexing methods

All the demultiplexing methods were built and run from a singularity image.

#### Demuxalot

*Demuxalot* [6] is a genotype reference-based single cell demultiplexing method. *Demualot* v0.2.0 was used in python v3.8.5 to annotate droplets. The likelihoods, posterior probabilities and most likely donor for each droplet were estimated using the *Demuxalot Demultiplexer.predict_posteriors* function. We also used *Demuxalot Demultiplexer.learn_genotypes* function to refine the genotypes before estimating the likelihoods, posterior probabilities and likely donor of each droplet with the refined genotypes as well.

#### Popscle

The Popscle v0.1-beta suite [16] for population genomics in single cell data was used for *Demuxlet* and *Freemuxlet* demultiplexing methods. The *popscle dsc-pileup* function was used to create a pileup of variant calls at known genomic locations from aligned sequence reads in each droplet with default arguments.

### Demuxlet

*Demuxlet* [3] is a SNP genotype reference-based single cell demultiplexing method. *Demuxlet* was run with a genotype error coefficient of 1 and genotype error offset rate of 0.05 and the other default parameters using the *popscle demuxlet* command from Popscle (v0.1-beta).

### Freemuxlet

*Freemuxlet* [16] is a SNP genotype reference-free single cell demultiplexing method. *Freemuxlet* was run with default parameters including the number of samples included in the pool using the *popscle freemuxlet* command from Popscle (v0.1-beta).

#### Dropulation

*Dropulation* [5] is a SNP genotype reference-based single cell demultiplexing method that is part of the *Drop-seq* software. *Dropulation* from *Drop-seq* v2.5.1 was implemented for this manuscript. In addition, the method for calling singlets and doublets was provided by the *Dropulation* developer and implemented in a custom R script available on Github and Zenodo (see “Availability of data and materials”).

#### ScSplit

*ScSplit* v1.0.7 [7] was downloaded from the *ScSplit* github and the recommended steps for data filtering quality control prior to running *ScSplit* were followed. Briefly, reads that had read quality lower than 10, were unmapped, were secondary alignments, did not pass filters, were optical PCR duplicates or were duplicate reads were removed. The resulting bam file was then sorted and indexed followed by freebayes to identify single nucleotide variants (SNVs) in the dataset. The resulting SNVs were filtered for quality scores greater than 30 and for variants present in the reference SNP genotype vcf. The resulting filtered bam and vcf files were used as input for the s*cSplit count* command with default settings to count the number of reference and alternative alleles in each droplet. Next the allele matrices were used to demultiplex the pool and assign cells to different clusters using the *scSplit run* command including the number of individuals (*-n*) option and all other options set to default. Finally, the individual genotypes were predicted for each cluster using the *scSplit genotype* command with default parameters.

#### Souporcell

*Souporcell* [4] is a SNP genotype reference-free single cell demultiplexing method. The *Souporcell* v1.0 singularity image was downloaded via instructions from the gihtub page. The *Souporcell* pipeline was run using the *souporcell_pipeline.py* script with default options and the option to include known variant locations (*--common_variants*).

#### Vireo

*Vireo* [2] is a single cell demultiplexing method that can be used with reference SNP genotypes or without them. For this assessment, *Vireo* was used with reference SNP genotypes. Per *Vireo* recommendations, we used model 1 of the *cellSNP* [20] version 0.3.2 to make a pileup of SNPs for each droplet with the recommended options using the genotyped reference genotype file as the list of common known SNP and filtered with SNP locations that were covered by at least 20 UMIs and had at least 10% minor allele frequency across all droplets. *Vireo* version 0.4.2 was then used to demultiplex using reference SNP genotypes and indicating the number of individuals in the pools.

### Doublet detecting methods

All doublet detecting methods were built and run from a singularity image.

#### DoubletDecon

*DoubletDecon* [9] is a transcription-based deconvolution method for identifying doublets. *DoubletDecon* version 1.1.6 analysis was run in R version 3.6.3. *SCTransform* [21] from Seurat [22] version 3.2.2 was used to preprocess the scRNA-seq data and then the *Improved_Seurat_Pre_Process* function was used to process the SCTransformed scRNA-seq data. Clusters were identified using Seurat function *FindClusters* with resolution 0.2 and 30 principal components (PCs). Then the *Main_Doublet_Decon* function was used to deconvolute doublets from singlets for six different rhops—0.6, 0.7, 0.8, 0.9, 1.0 and 1.1. We used a range of rhop values since the doublet annotation by *DoubletDecon* is dependent on the rhop parameter which is selected by the user. The rhop that resulted in the closest number of doublets to the expected number of doublets was selected on a per-pool basis and used for all subsequent analysis. Expected number of doublets were estimated with the following equation:

$$D=\frac{{N}^{2}\times 0.008}{1000}$$

where *N* is the number of droplets captured and *D* is the number of expected doublets.

#### DoubletDetection

*DoubletDetection* [14] is a transcription-based method for identifying doublets. *DoubletDetection* version 2.5.2 analysis was run in python version 3.6.8. Droplets without any UMIs were removed before analysis with *DoubletDetection*. Then the *doubletdetection.BoostClassifier* function was run with 50 iterations with *use_phenograph* set to False and *standard_scaling* set to True. The predicted number of doublets per iteration was visualised across all iterations and any pool that did not converge after 50 iterations, it was run again with increasing numbers of iterations until they reached convergence.

#### DoubletFinder

*DoubletFinder* [10] is a transcription-based doublet detecting method. *DoubletFinder* version 2.0.3 was implemented in R version 3.6.3. First, droplets that were more than 3 median absolute deviations (mad) away from the median for mitochondrial per cent, ribosomal per cent, number of UMIs or number of genes were removed per developer recommendations. Then the data was normalised with SCTransform followed by cluster identification using *FindClusters* with resolution 0.3 and 30 principal components (PCs). Then, pKs were selected by the pK that resulted in the largest BC_MVN_ as recommended by DoubletFinder. The pK vs BC_MVN_ relationship was visually inspected for each pool to ensure an effective BC_MVN_ was selected for each pool. Finally, the homotypic doublet proportions were calculated and the number of expected doublets with the highest doublet proportion were classified as doublets per the following equation:

$$D=\frac{{N}^{2}\times 0.008}{1000}$$

where *N* is the number of droplets captured and *D* is the number of expected doublets.

#### ScDblFinder

*ScDblFinder* [11] is a transcription-based method for detecting doublets from scRNA-seq data. *ScDblFinder* 1.3.25 was implemented in R version 4.0.3. *ScDblFinder* was implemented with two sets of options. The first included implementation with the expected doublet rate as calculated by:

$$R=\frac{{N}\times 0.008}{1000}$$

where *N* is the number of droplets captured and *R* is the expected doublet rate. The second condition included the same expected number of doublets and included the doublets that had already been identified by all the demultiplexing methods.

#### Scds

*Scds* [12] is a transcription-based doublet detecting method. Scds version 1.1.2 analysis was completed in R version 3.6.3. *Scds* was implemented with the *cxds* function and *bcds* functions with default options followed by the *cxds_bcds_hybrid* with *estNdbl* set to TRUE so that doublets will be estimated based on the values from the *cxds* and *bcds* functions.

#### Scrublet

*Scrublet* [13] is a transcription-based doublet detecting method for single-cell RNA-seq data. *Scrublet* was implemented in python version 3.6.3. *Scrublet* was implemented per developer recommendations with at least 3 counts per droplet, 3 cells expressing a given gene, 30 PCs and a doublet rate based on the following equation:

$$R=\frac{{N}\times 0.008}{1000}$$

where *N* is the number of droplets captured and *R* is the expected doublet rate. Four different minimum number of variable gene percentiles: 80, 85, 90 and 95. Then, the best variable gene percentile was selected based on the distribution of the simulated doublet scores and the location of the doublet threshold selection. In the case that the selected threshold does not fall between a bimodal distribution, those pools were run again with a manual threshold set.

#### Solo

*Solo* [15] is a transcription-based method for detecting doublets in scRNA-seq data. *Solo* was implemented with default parameters and an expected number of doublets based on the following equation:

$$D=\frac{{N}^{2}\times 0.008}{1000}$$

where *N* is the number of droplets captured and *D* is the number of expected doublets. *Solo* was additionally implemented in a second run for each pool with the doublets that were identified by all the demultiplexing methods as known doublets to initialize the model.

### In silico pool generation

Cells that were identified as singlets by all methods were used to simulate pools. Ten pools containing 2, 4, 8, 16, 32, 64 and 128 individuals were simulated assuming a maximum 20% doublet rate as it is unlikely researchers would use a technology that has a higher doublet rate. The donors for each simulated pool were randomly selected using a custom R script which is available on Github and Zenodo (see ‘Availability of data and materials’). A separate bam for the cell barcodes for each donor was generated using the *filterbarcodes* function from the *sinto* package (v0.8.4). Then, the *GenerateSyntheticDoublets* function provided by the *Drop-seq* [5] package was used to simulate new pools containing droplets with known singlets and doublets.

Twenty-one total pools—three pools from each of the different simulated pool sizes (2, 4, 8, 16, 32, 64 and 128 individuals) —were used to simulate different experimental scenarios that may be more challenging for demultiplexing and doublet detecting methods. These include simulating higher ambient RNA, higher mitochondrial percent, decreased read coverage and imbalanced donor proportions as described subsequently.

#### High ambient RNA simulations

Ambient RNA was simulated by changing the barcodes and UMIs on a random selection of reads for 10, 20 or 50% of the total UMIs. This was executed with a custom R script that is available in Github and Zenodo (see ‘Availability of data and materials’).

#### High mitochondrial percent simulations

High mitochondrial percent simulations were produced by replacing reads in 5, 10 or 25% of the randomly selected cells with mitochondrial reads. The number of reads to replace was derived from a normal distribution with an average of 30 and a standard deviation of 3. This was executed with a custom R script available in Github and Zenodo (see ‘Availability of data and materials’).

#### Imbalanced donor simulations

We simulated pools that contained uneven proportions of the donors in the pools to identify if some methods are better at demultiplexing pools containing uneven proportions of each donor in the pool. We simulated pools where 50, 75 or 95% of the pool contained cells from a single donor and the remainder of the pool was even proportions of the remaining donors in the pool. This was executed with a custom R script available in Github and Zenodo (see ‘Availability of data and materials’).

#### Decrease read coverage simulations

Decreased read coverage of pools was simulated by down-sampling the reads by two-thirds of the original coverage.

### Classification annotation

#### Demultiplexing methods

Demultiplexing methods classifications were considered correct if the droplet annotation (singlet or doublet) and the individual annotation was correct. If the droplet type was correct but the individual annotation was incorrect (i.e. classified as a singlet but annotated as the wrong individual), then the droplet was incorrectly classified.

#### Doublet detecting methods

Doublet detecting methods were considered to have correct classifications if the droplet annotation matched the known droplet type.

### Analyses

All downstream analyses were completed in R version 4.0.2.

### Supplementary Information

**Additional file 1: Supplementary Tables and legends.**

**Additional file 2: Supplementary Figures and legends.**

**Additional file 3.** Review history.

## Data Availability

All data used in this manuscript is publicly available. The PBMC data is available on GEO (Accession: GSE196830) [23] as originally described in [17]. The fibroblast data is available on ArrayExpress (Accession Number: E-MTAB-10060) [24] and as originally described in [18]. The code used for the analyses in this manuscript are provided on Github (https://github.com/powellgenomicslab/Demuxafy_manuscript/tree/v4) and Zenodo (https://zenodo.org/records/10813452) under an MIT Open Source License [25, 26]. Demuxafy is provided as a package with source code available on Github (https://github.com/drneavin/Demultiplexing_Doublet_Detecting_Docs) and instructions on ReadTheDocs (https://demultiplexing-doublet-detecting-docs.readthedocs.io/en/latest/) under an MIT Open Source License [27]. Demuxafy is also available on Zenodo with the link https://zenodo.org/records/10870989[28].
