# Supplementary material for: Demuxafy: improvement in droplet assignment by integrating multiple single-cell demultiplexing and doublet detection methods
Source: Genome Biol. 2024 Apr 15;25:94. doi: 10.1186/s13059-024-03224-8 (PMC11020463; doi:10.1186/s13059-024-03224-8)
Supplement: Supplementary file 2 — Additional file 2: Supplementary Figures and legends. [file 13059_2024_3224_MOESM2_ESM.docx]

**Supplementary Materials**

***Demuxafy:* improvement in droplet assignment by integrating multiple single-cell demultiplexing and doublet detection methods**

Drew Neavin^1,2^*, Anne Senabouth^1^, Himanshi Arora^1,3^, Jimmy Tsz Hang Lee^4,5^, Aida Ripoll-Cladellas^6^, sc-eQTLGen Consortium, Lude Franke^7^, Shyam Prabhakar^8,9,10^, Chun Jimmie Ye^11,12,13,14^, Davis J. McCarthy^15,16^, Marta Melé^6,17^, Martin Hemberg^5,18^, Joseph E. Powell^1,19^*

^1^Garvan-Weizmann Centre for Cellular Genomics, Garvan Institute for Medical Research, Darlinghurst, NSW, Australia

^2^@drneavin

^3^Present address: Statewide Genomics at NSW Health Pathology, Sydney, NSW, Australia

^4^Wellcome Sanger Institute, Wellcome Genome Campus, Hinxton, UK

^5^@thjimmylee

^6^Life Sciences Department, Barcelona Supercomputing Center, Barcelona, Catalonia, Spain

^7^Department of Genetics, University of Groningen, University Medical Center Groningen, Groningen, The Netherlands

^8^Spatial and Single Cell Systems Domain, Genome Institute of Singapore (GIS), Agency for Science, Technology and Research (A*STAR), Singapore, Republic of Singapore

^9^⁠Population and Global Health, Lee Kong Chian School of Medicine, Nanyang Technological University, Singapore, Republic of Singapore

^10^Cancer Science Institute of Singapore, National University of Singapore, Singapore, Republic of Singapore

^11^Bakar Institute for Computational Health Sciences, University of California, San Francisco, San Francisco, CA, USA

^12^Institute for Human Genetics, University of California, San Francisco, San Francisco, CA, USA

^13^Division of Rheumatology, Department of Medicine, University of California, San Francisco, San Francisco, CA, USA

^14^Chan Zuckerberg Biohub, San Francisco, CA, USA

^15^Bioinformatics and Cellular Genomics, St Vincent’s Institute of Medical Research, Fitzroy, Australia

^16^Melbourne Integrative Genomics, School of BioSciences–School of Mathematics & Statistics, Faculty of Science, University of Melbourne, Melbourne, Australia

^17^@marta_mele_m

^18^Present address: The Gene Lay Institute of Immunology and Inflammation, Brigham and Women’s Hospital and Harvard Medical School, Boston, MA, USA

^19^UNSW Cellular Genomics Futures Institute, University of New South Wales, Kensington, NSW, Australia

*Correspondence:

d.neavin@garvan.org.au;

j.powell@garvan.org.au

S


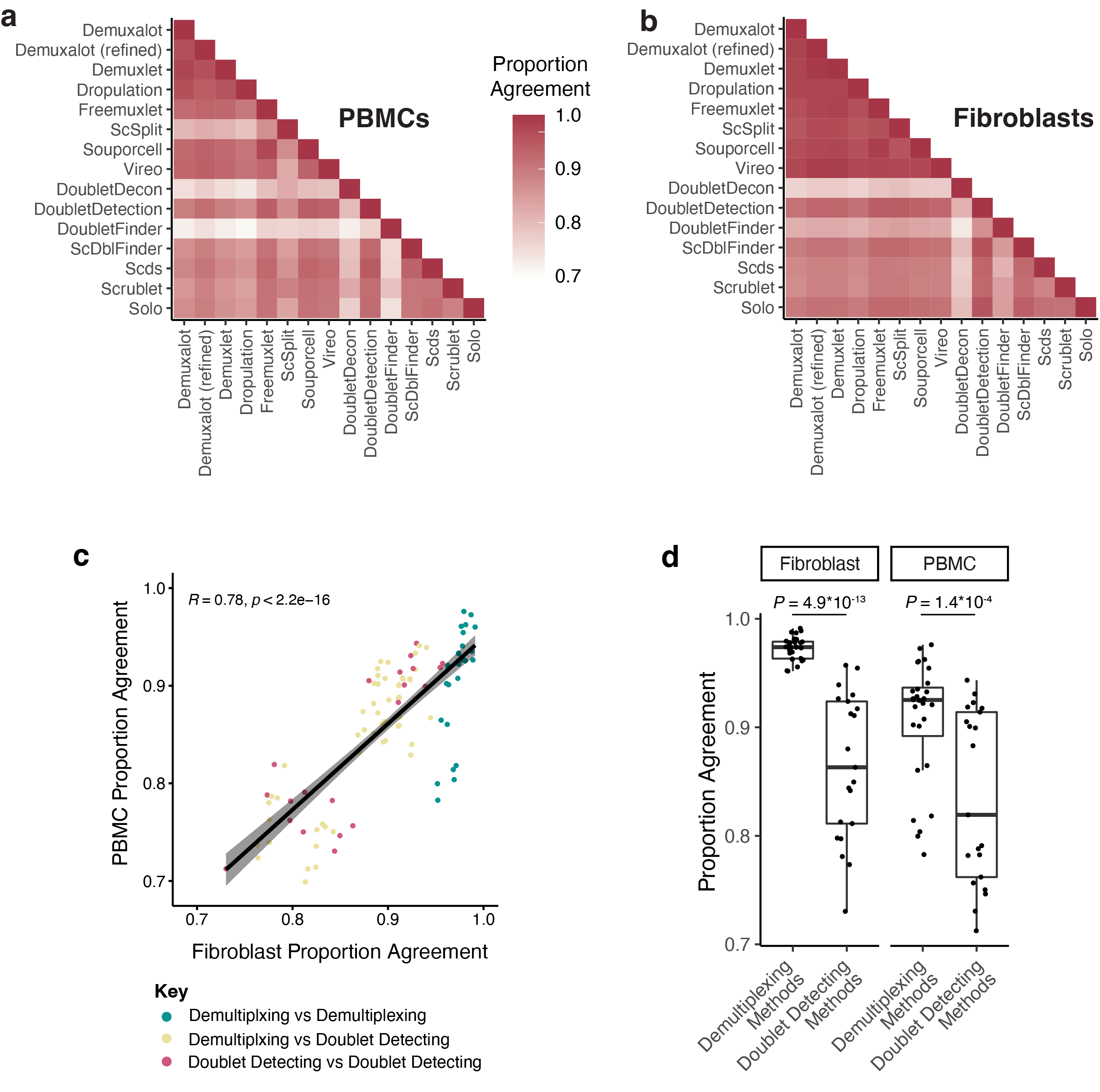


**Fig S1:** **Association of Method Correlations Between PBMC and Fibroblast Cell Types**. **a-b**) Heatmap of agreement of droplet classifications between different methods for the PBMCs (**a**) and fibroblasts (**b**). **c**) The pairwise correlations between the demultiplexing and doublet detecting methods were tested for association between the PBMC and fibroblast cell types. The colours indicate which method types are being compared in the correlation. Spearman correlation was used to test the relationship between the PBMC and Fibroblast correlations. **d**) Comparison of the correlations between the demultiplexing and doublet detecting methods. Distributions were tested with a one-sided Wilcoxon rank-sum test.

**
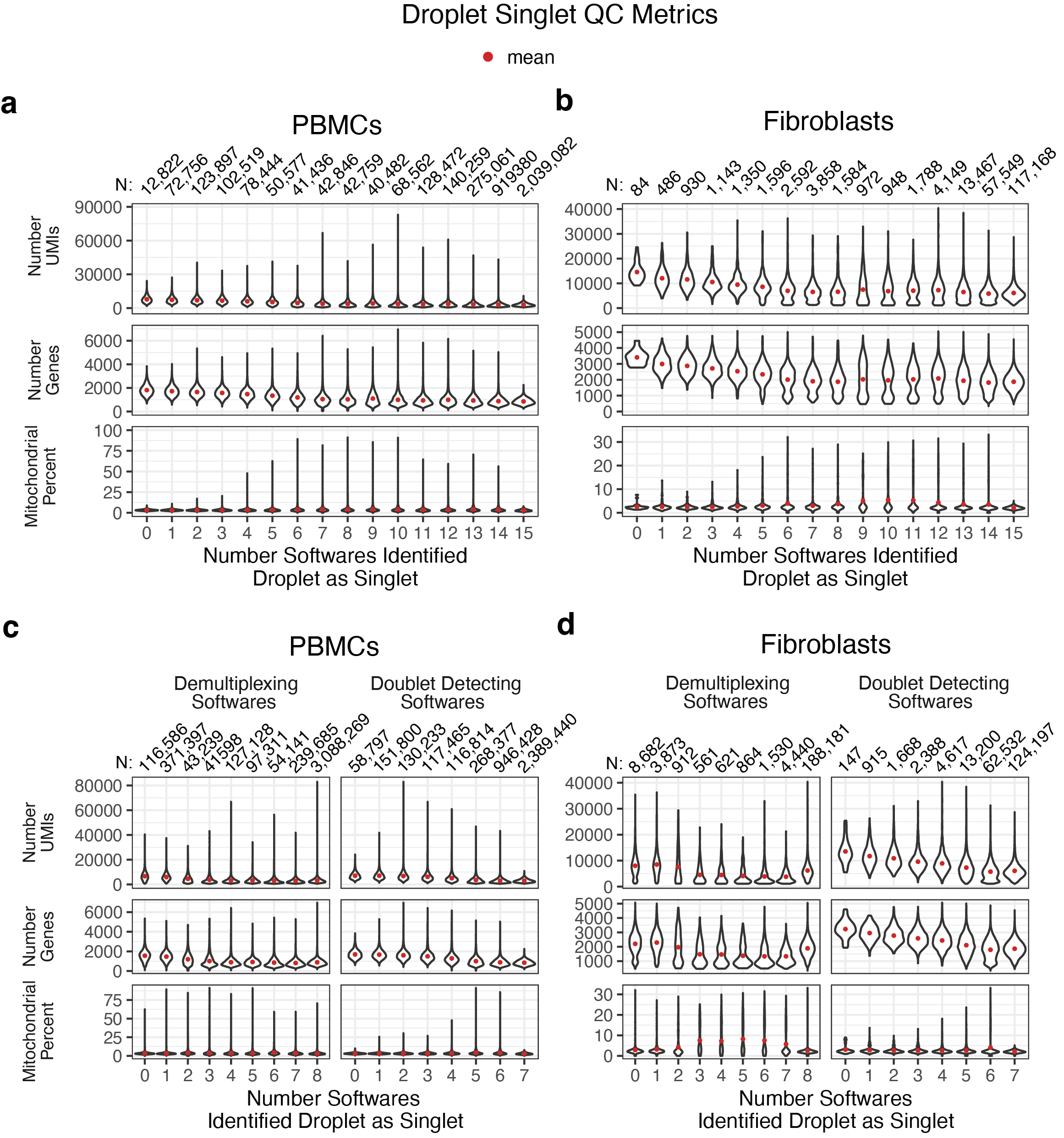
 Fig S2: Cell Type Metrics for Droplets Classified as Singlets by Different Method Combinations.** The distribution of mitochondrial percent, number of unique molecular identifiers (UMIs) and number of genes is demonstrated for droplets classified as singlets by different combinations of softwares in PBMCs (**a**) and Fibroblasts (**b**). This is further demonstrated for the eight demultiplexing softwares and seven doublet detecting softwares separately for the PBMCs (**c**) and Fibroblasts (**d**)


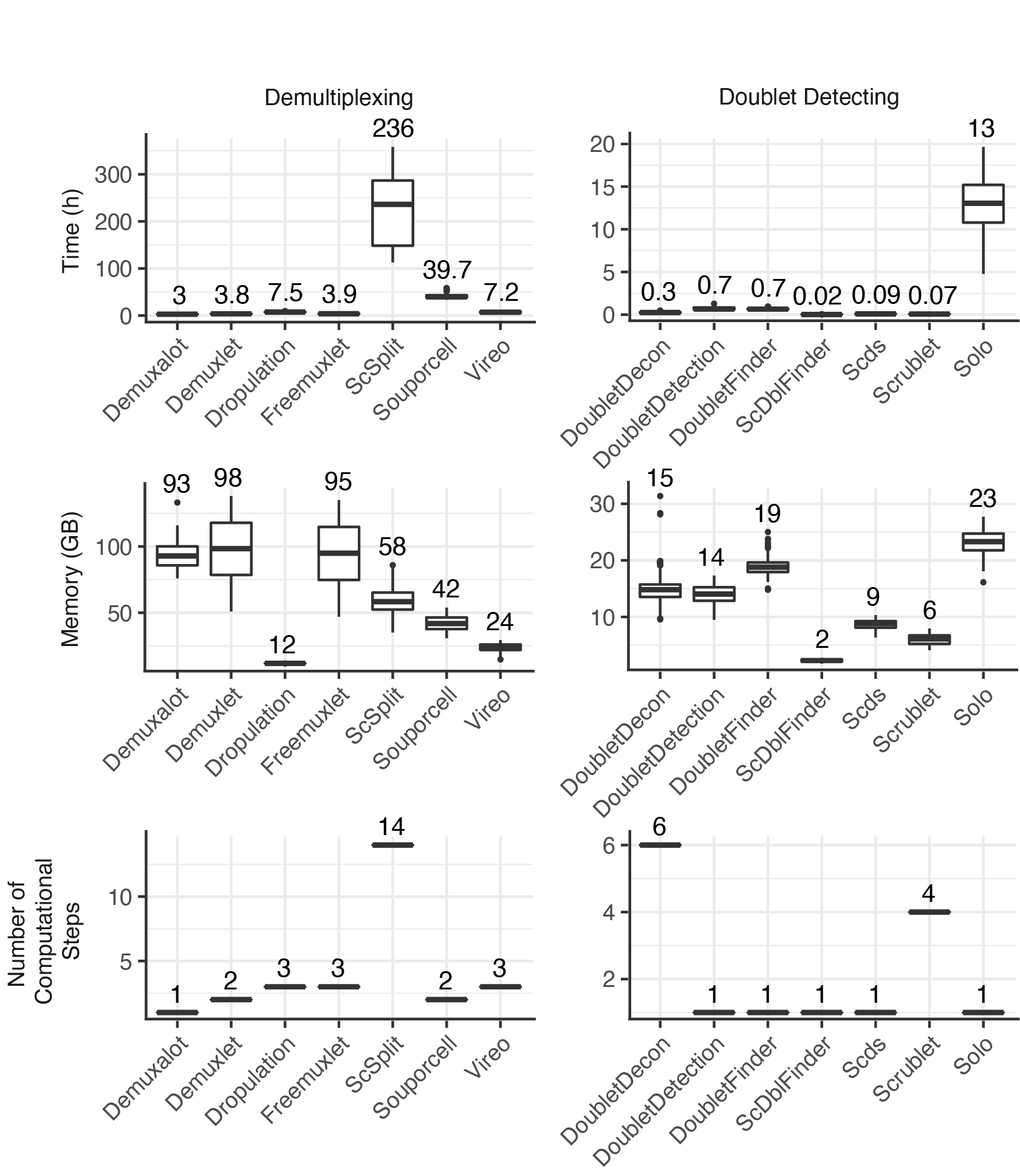


**Fig S3: The time, memory and number of computational steps for each method.** The time in hours (h), the memory in gigabytes (GB) and the number of user-required computational steps for each of the demultiplexing (left) and doublet detecting methods (right).


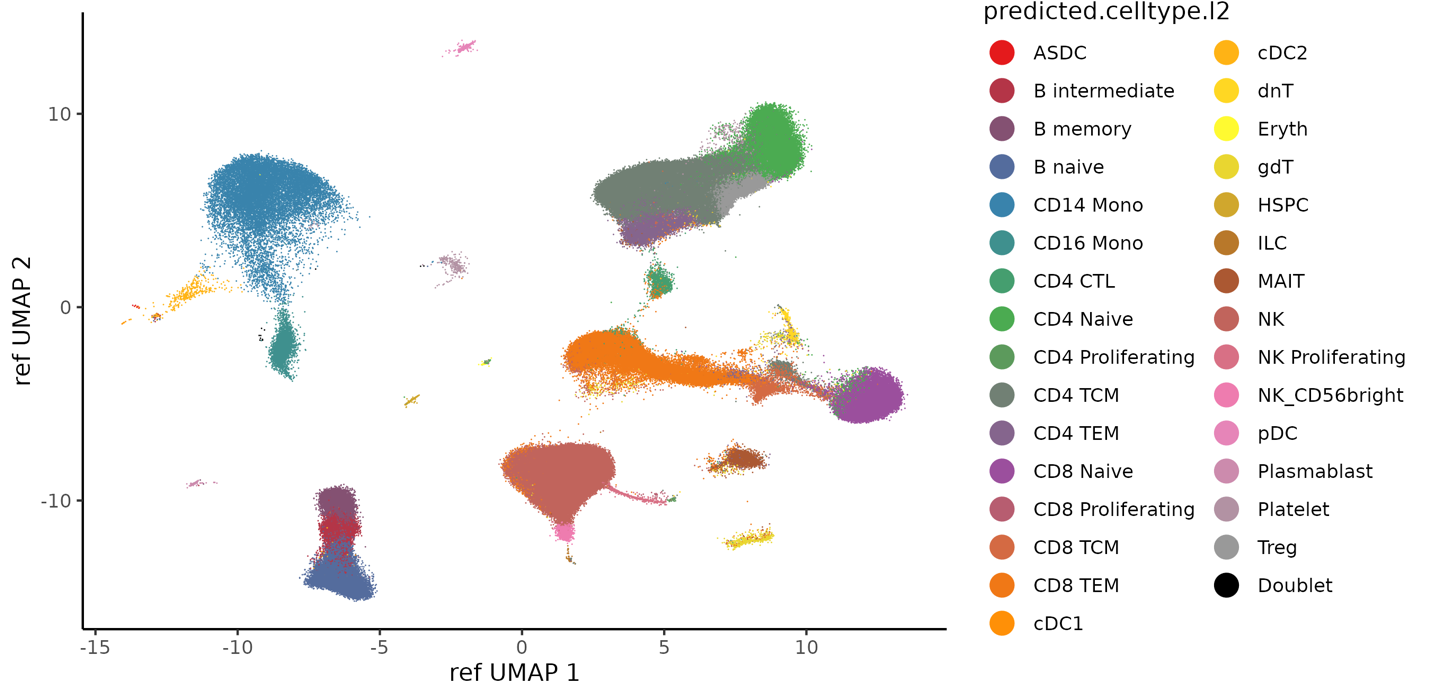


**Fig S4: Cell Type Annotation of droplets classified as singlets by all methods.** The cell type annotation of the PBMCs that were all classified as singlets by all the demultiplexing and doublet detecting methods using the Azimuth PBMC reference.


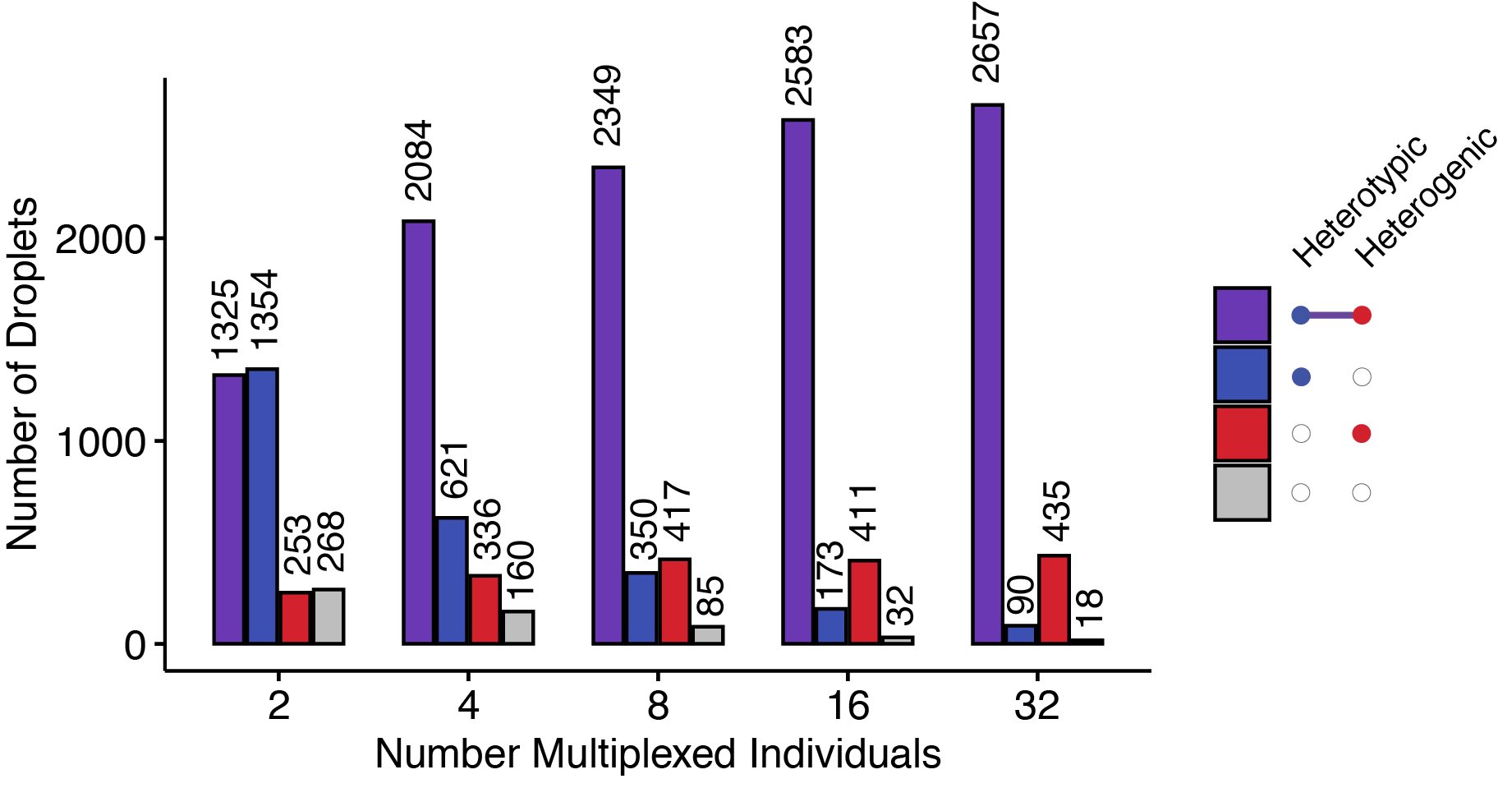


**Fig S5: Number of different doublet types assuming 20,000 droplets captured.**  The number of different types of doublets would be in pools that multiplexed 2, 4, 8, 16 and 32, assuming 20,000 droplets were captured.

**Fig S6: Each demultiplexing method's percentage of each cell type annotated correctly.** The percentage of droplets correctly classified by each demultiplexing method for each cell type across different multiplexed pool sizes. The number of each cell type per 10,000 droplets is shown in grey beneath the cell type name. N = 10 for each group.

 **Fig S7: The percentage of each doublet detecting method’s cell type annotated correctly.** The percentage of droplets correctly classified by each doublet detecting method for each cell type across different multiplexed pool sizes. The number of each cell type per 10,000 droplets is shown in grey beneath the cell type name. N = 10 for each group.

**Fig S8: Percent Incorrect Droplet Classifications.** The percent of droplets incorrectly classified for each method across multiple multiplexed pool sizes. For demultiplexing methods, the droplet type and individual assignment had to be correct for the overall assignment to be considered valid.

**
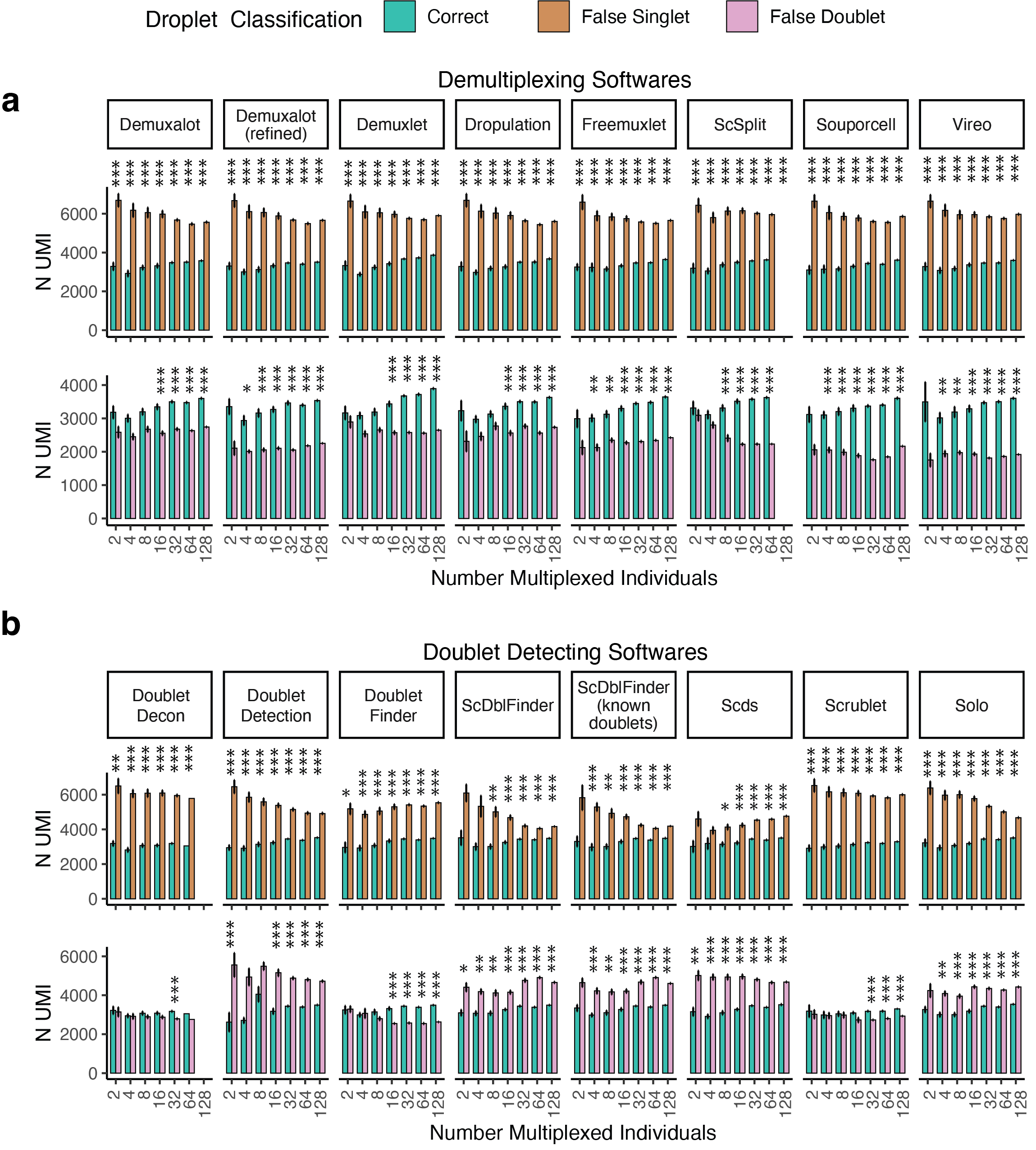
 Fig S9: Number of UMIs for False Doublets and Singlets Compared to Correctly Classified Droplets.** **a**) The false singlet droplets had higher UMI counts than the correctly identified droplets for all demultiplexing methods for all pool sizes. The false doublets demonstrated lower UMI counts for most methods and pool sizes. The exceptions were smaller pools. **b**) Similar to the demultiplexing methods, the falsely identified singlets by doublet detecting methods had higher UMI counts than the correctly classified droplets. Contrary to the demultiplexing methods, the droplets falsely classified as doublets by the doublet detecting methods typically had higher UMI counts with a few exceptions: *DoubletDecon* pool size 32, and larger pools for *Doublet Finder* and *Scrublet.* The difference in the number of UMIs between correct and incorrect droplet annotation was tested with a student’s *t*-test, and *P*-values were corrected for multiple testing with the Bonferroni method. * 0.01 < *P* < 0.05; ** 0.001 < *P* < 0.01; *** *P* < 0.001.

**
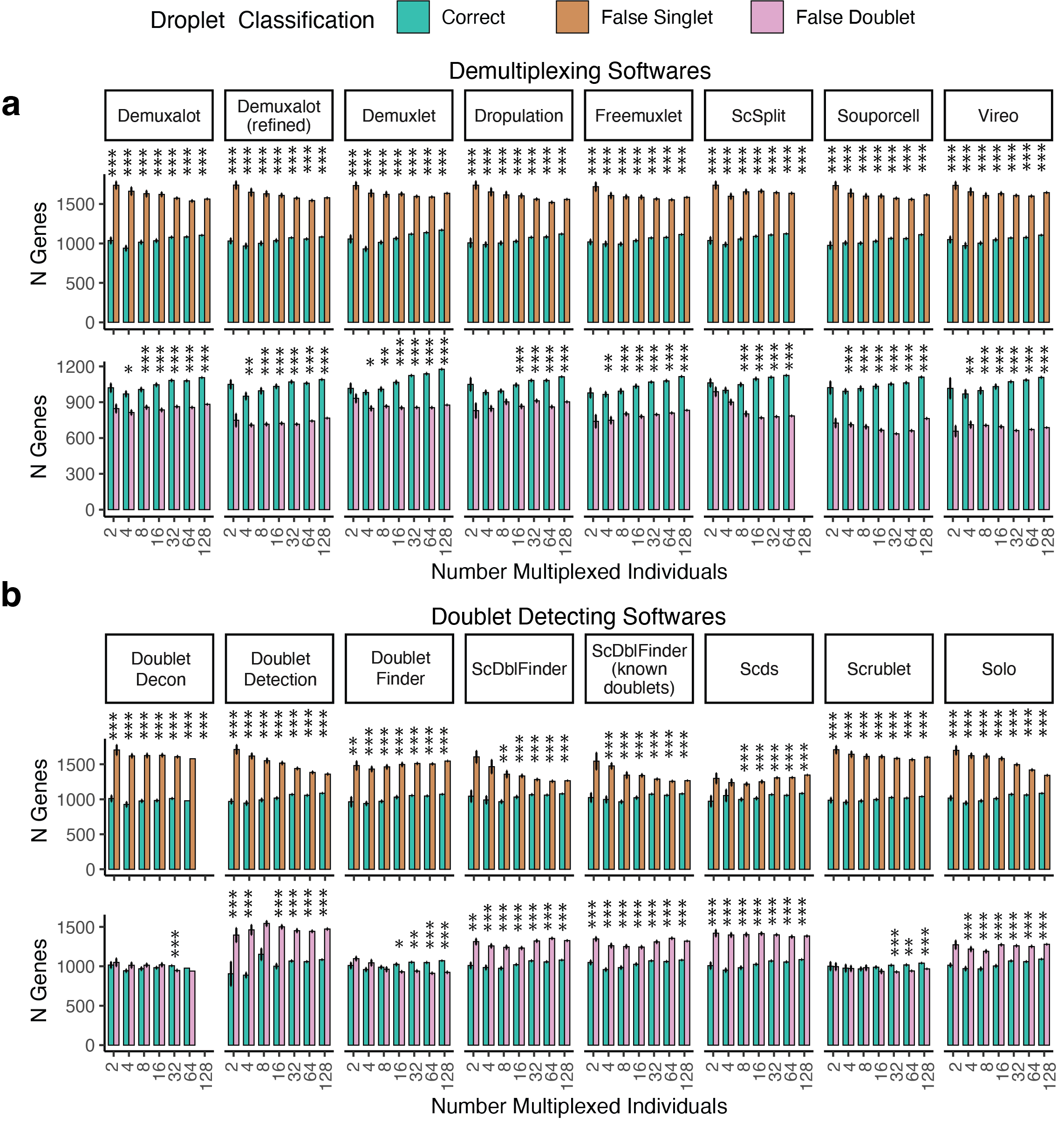
 Fig S10: Number of Genes in False Doublets and Singlets Compared to Correctly Classified Droplets.** **a**) The false singlet droplets had higher gene counts than the correctly identified droplets for all demultiplexing methods for all pool sizes. The false doublets demonstrated lower gene counts for most methods and pool sizes. The exceptions were smaller pools. **b**) Like the demultiplexing methods, the falsely identified singlets by doublet detecting methods had higher gene counts than the correctly classified droplets. The false singlets identified by *DoubletDetection*, *scDblFinder*, *scDblFinder* with known doublets, *Scds* and all had higher gene counts than the correct droplets. Some of the larger pools demonstrated that the false singlets had fewer genes than the correctly identified droplets by *DoubletFinder*, *DoubletDecon* and *Scrublet*. The difference in genes between correct and incorrect droplet annotation was tested with a student’s t-test, and P-values were corrected for multiple testing using the Bonferroni method. * 0.01 < *P* < 0.05; ** 0.001 < *P* < 0.01; *** *P* < 0.001.

**
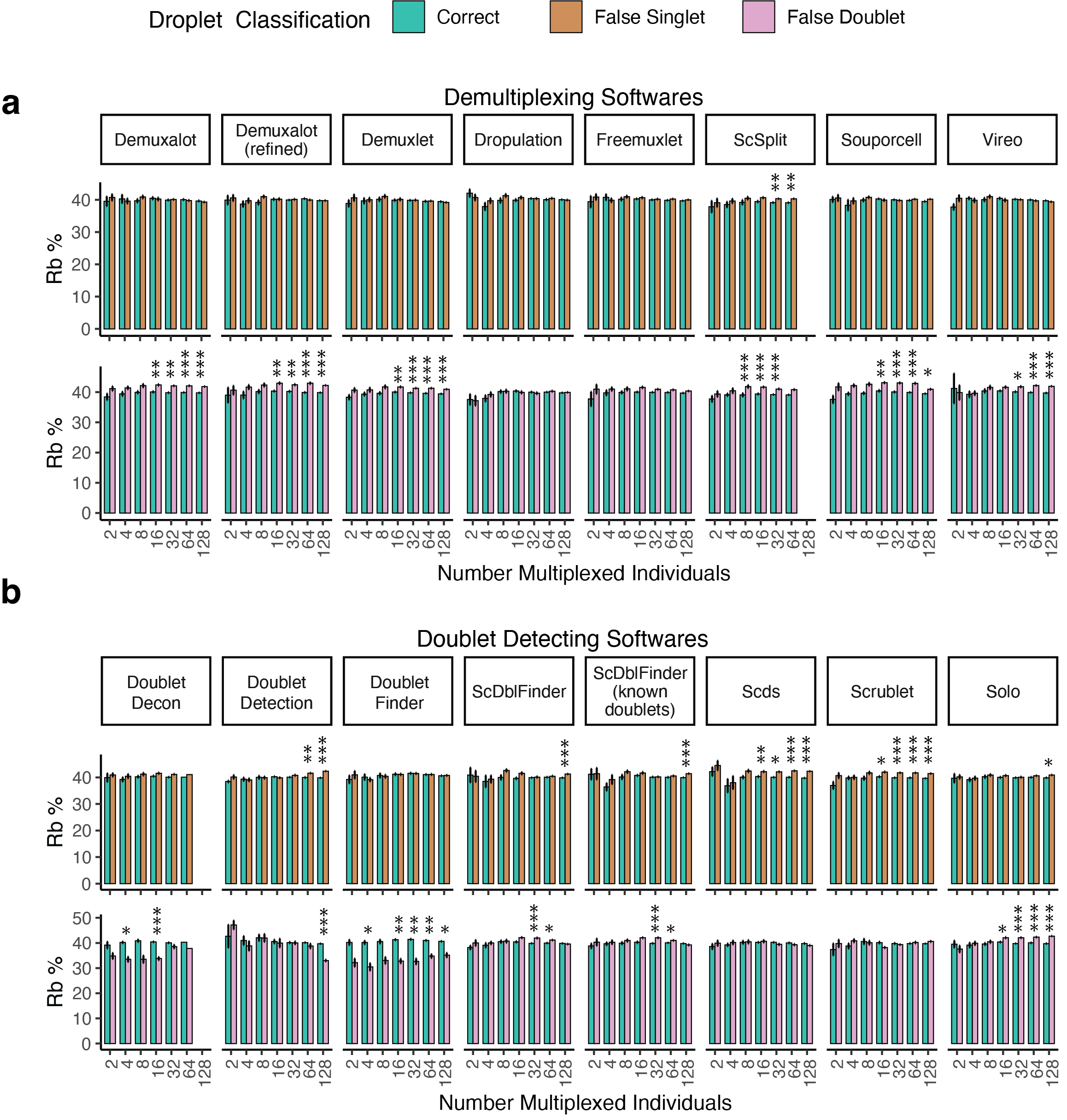
 Fig S11: Ribosomal Percent in False Doublets and Singlets Compared to Correctly Classified Droplets.** **a**) The ribosomal percent in false singlet droplets were consistent with the correctly identified droplets for the demultiplexing methods except for *scSplit* which demonstrated small but significant differences. The false doublets demonstrated small but significant increases in ribosomal percent for larger pool sizes for *Demuxalot*, *Demuxalot (refinded)*, *Demuxlet*, *ScSplit*, *Souporcell* and *Vireo*. **b**) The doublet detecting methods demonstrated small but significantly higher ribosomal percent in the false doublets than the correctly classified droplets. However, this effect was not as noticeable in *DoubletDecon* or *DoubletFinder*. The false doublets were less consistent across different doublet detecting methods. Some methods demonstrated significantly lower ribosomal percent in the false doublets (*i.e.* *DoubletDecon*, *DoubletDetection* and *DoubletFinder*). In contrast, other methods demonstrated significantly higher ribosomal percent in the falsely identified doublets (*i.e.* *ScDblFinder*, *ScDblFinder* with known doublets and *Solo*). The difference in genes between correct and incorrect droplet annotation was tested with a student’s t-test, and *P*-values were corrected for multiple testing using the Bonferroni method. * 0.01 < *P* < 0.05; ** 0.001 < *P* < 0.01; *** *P* < 0.001.

**
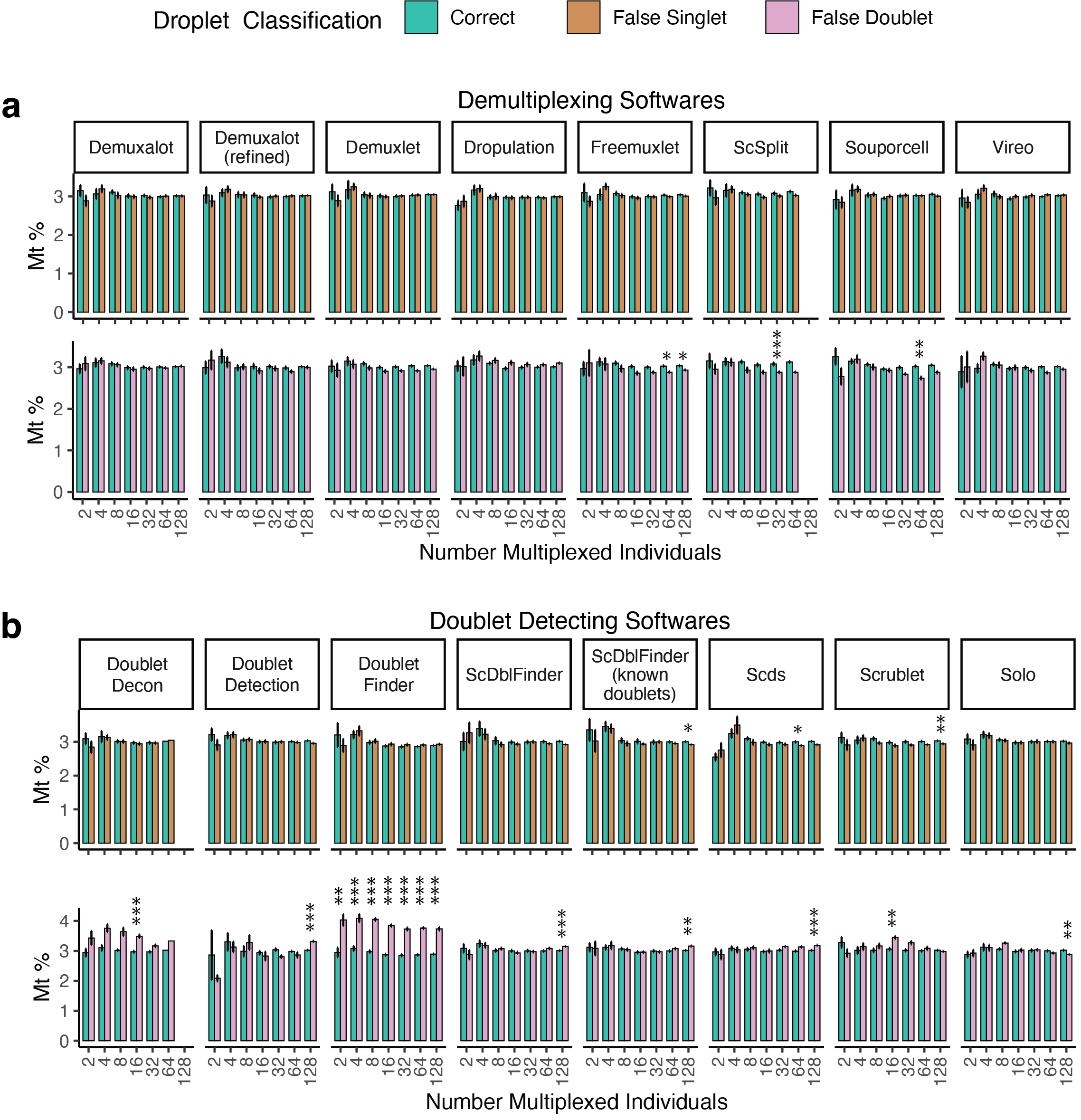
 Fig S12: Mitochondrial Percent in False Doublets and Singlets Compared to Correctly Classified Droplets.** **a**) None of the demultiplexing methods demonstrated difference in the mitochondrial percent in the false singlets than the correctly classified droplets. However, the false doublets had lower mitochondrial percent for a handful of pools for *Freemuxlet*, *ScSplit* and *Souporcell*. **b**) The doublet detecting methods demonstrated small but significantly lower mitochondrial percent for large pool sizes for false doublets by *ScDblFinder* (known doublets), *Scds* and *Scrublet*. The false doublets identified by most doublet detecting methods had higher mitochondrial percent than the correctly classified droplets which was especially consistent for *DoubletFinder*. However, the pool of 128 donors demonstrated a small but significant decrease in the mitochondrial percent for Solo. The difference in genes between correct and incorrect droplet annotation was tested with a student’s t-test, and *P*-values were corrected for multiple testing using the Bonferroni method. * 0.01 < *P* < 0.05; ** 0.001 < *P* < 0.01; *** *P* < 0.001.

**Fig S13: Influence of downsampling, additional ambient RNA percent, additional mitochondrial percent and donor spiking on method performance.** The performance of each method for each simulated condition was assessed with the MCC (left) and balanced accuracy (right). **a)** Downsampling the number of reads per cell by 50% resulted in a consistent decrease in performance. The degree of this effect is different for each method with less overall impact on the doublet detecting methods. **b)** Simulating additional ambient RNA into each droplet decreased the performance of most methods. This effect was more extreme for the demultiplexing methods – especially in the pools containing larger numbers of donors. Ambient RNA had less of an impact on the performance of the doublet detecting methods and was only noticeable for some of the methods for pools containing at least 32 donors. **c)** additional mitochondrial RNA did not influence the performance of each of the methods. **d)** spiking pools with a larger proportion of a single individual (0.5 or 0.75) resulted in significantly decreased performance for all the demultiplexing methods. Michael’s Correlation Coefficient (MCC)

**

 Fig S14: Comparison of Demuxafy to Chord.** **a)** Demuxafy resulted in higher Michael’s Correlation Coefficient (MCC) in all but 2 comparisons (one pool containing two donors and one containing four donors). This is more evident in the larger pools containing more donors and more droplets. **b)** This difference in MCC is due to an increased proportion of droplets falsely classified as doublets and singlets which had a reciprocal decrease in the proportion of droplets correctly classified as singlets and doublets. Only one group was able to be investigated for 128 donors because chord was unable to process the other two pools.


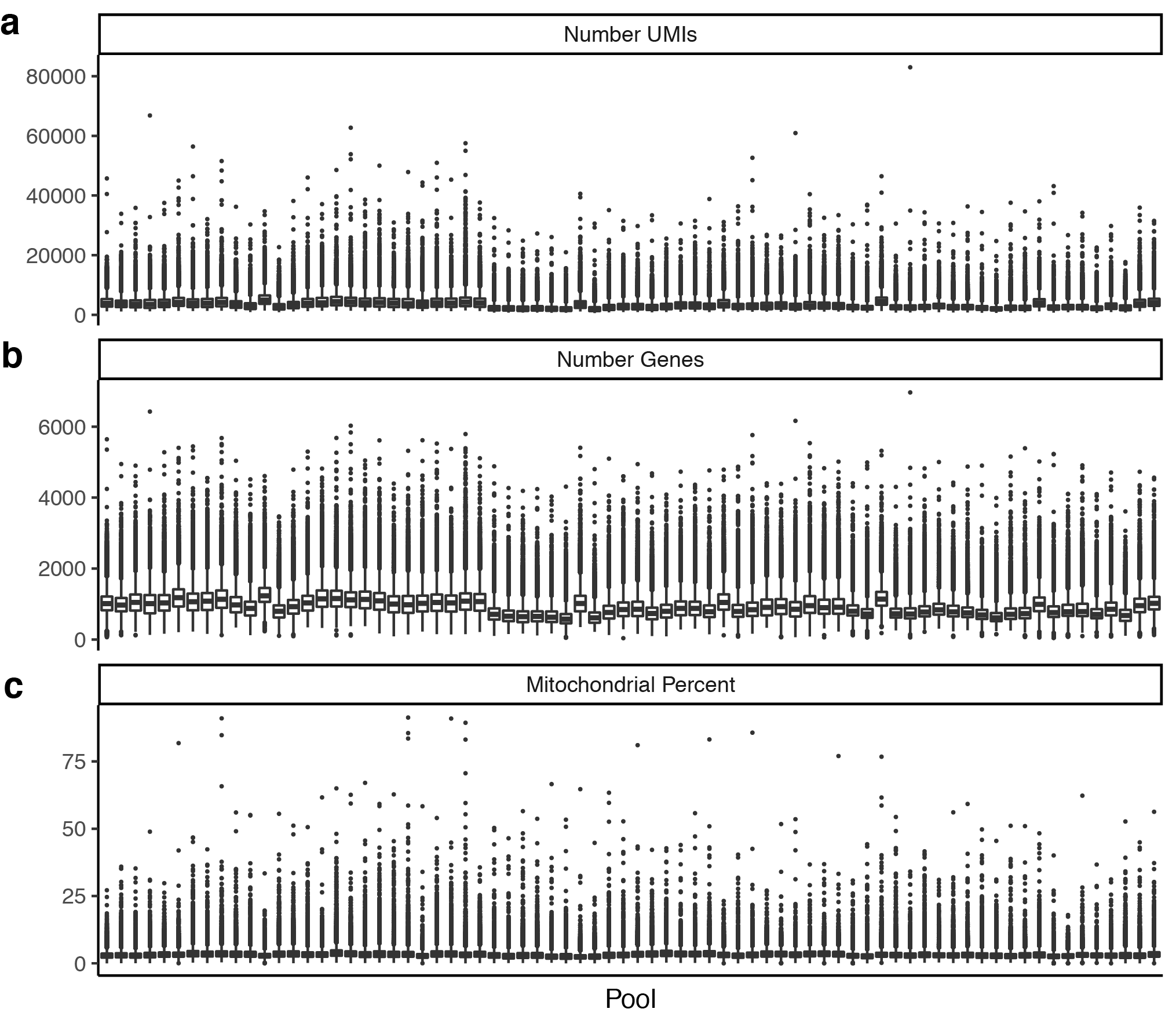


**Fig S15: Quality Control Metrics of the PBMC pools.** The number of UMIs (**a**), number of genes (**b**) and mitochondrial per cent (**c**) per pool for the peripheral blood mononuclear cell (PBMC) dataset.


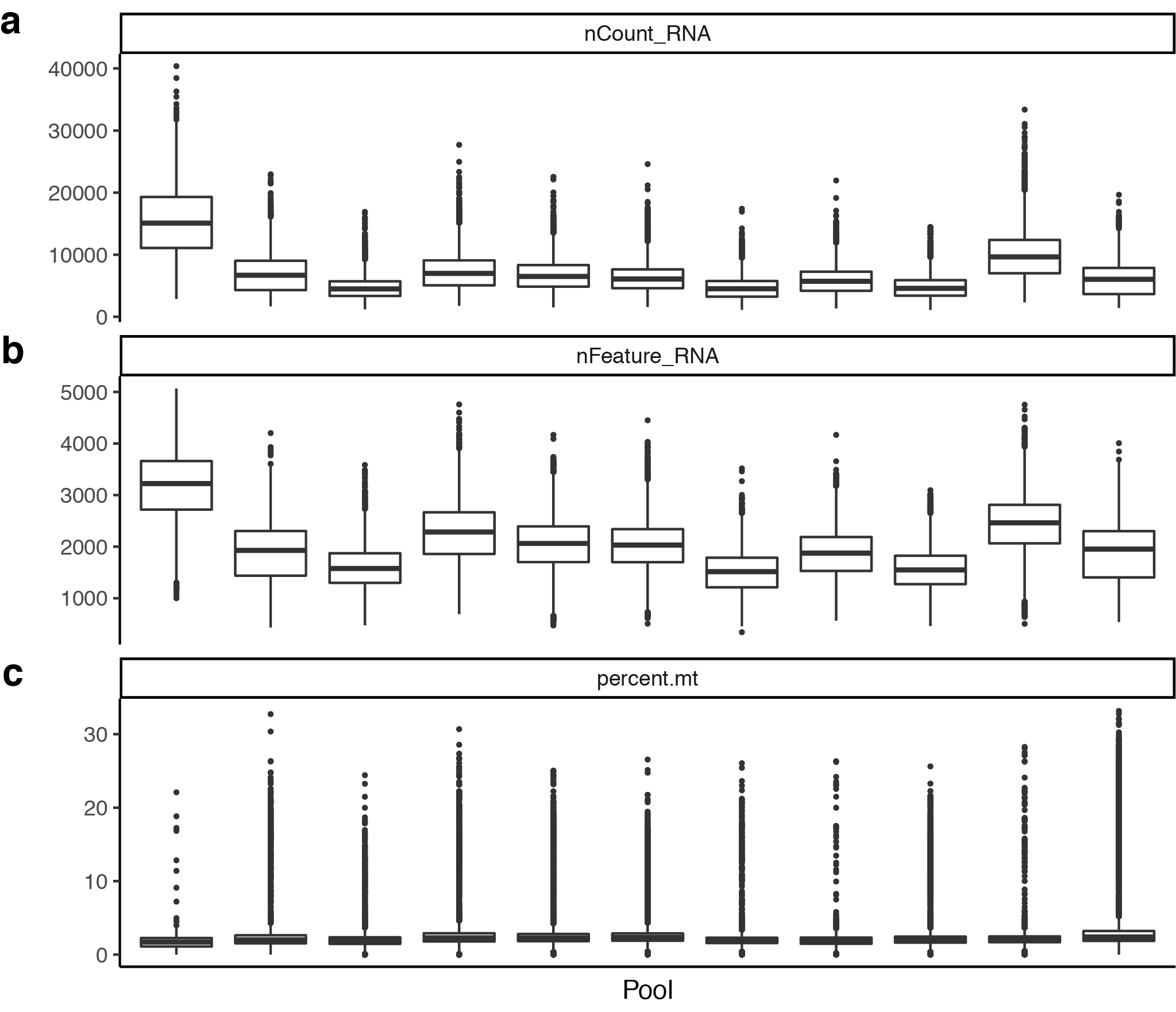


**Fig S16: Quality Control Metrics of the fibroblast pools.** The number of UMIs (**a**), number of genes (**b**) and mitochondrial per cent (**c**) per pool for the fibroblast dataset.
