# Supplementary material for: Demuxafy: improvement in droplet assignment by integrating multiple single-cell demultiplexing and doublet detection methods
Source: Genome Biol. 2024 Apr 15;25:94. doi: 10.1186/s13059-024-03224-8 (PMC11020463; doi:10.1186/s13059-024-03224-8)
Supplement: Supplementary file 3 — Additional file 3. Review history. [file 13059_2024_3224_MOESM3_ESM.docx]

Review History

**First round of review**

**Reviewer 1**

**Were you able to assess all statistics in the manuscript, including the appropriateness of statistical tests used?**

No.

**Were you able to directly test the methods?**

Yes.

**Comments to author:**

In the manuscript, the authors compare the performance of doublet and singlet annotation of current demultiplexing and doublet detection methods, focusing on large scRNA-Seq datasets generated from pooled cells from multiple individuals. By benchmarking the performance of the methods in two real datasets and simulated datasets in sillico, the authors conclude that it is unreliable to apply only one type of method on this type of data. Therefore, the authors integrate selected tools into a framework Demuxafy to improve the droplet assignment by intersectional approach. The authors also provide a brief guidance of the tool combinations when facing different experimental design.

While multiple tools are available for genetic demultiplexing, this is the first work seen benchmarking these tools. Plus, the authors raise a good point that demultiplexing methods and doublet detection methods are complementary in droplet assignment when genetically mixed cells are pooled and sequenced. However, an alternative strategy of integrating two types of methods should be considered. Due to the nature of design, demultiplexing methods are good at dealing with heterogenic doublets while doublet detection ones are good at detecting heterotypic doublets. Therefore, the modules integrating best performed demultiplexing methods and integrating best performed doublet detection methods can be included in the framework separately. When dealing with large scRNA-Seq datasets from genetically mixed cells, the sequential application of two modules can be employed to achieve better result. This will also make the framework more flexible. This strategy should be tested or at least discussed.

Specific points:

1. The authors ignore the previous comprehensive benchmarking report of doublet detection methods (Cell Syst. 2021 Feb 17;12(2):176-194.e6), which shows that DoubletFinder has the overall best performance. However, this method is not integrated in the framework as shown in Figure 5. The authors should justify the reason.

2. Similarly, the selection of the four demultiplexing tools into the framework is not justified.

3. In Figure 2a-b, it is unclear what does the agreement mean and how the score is generated. It is specifically obscure that the authors state the agreement is calculated in different ways when demultiplexing methods are compared vs. when doublet detection methods are compared. If so, it is inappropriate to have them in the same heatmap. It shows that DoubletDecon and DoubletFinder have clear disagreements with other methods in both datasets. Does it mean one or both methods are not reliable?

4. In Supplementary Figure 1, the authors show the correlation of every two methods. It demonstrates a wide range of correlation in doublet detection methods. The detailed correlation results should be included in a table.

5. The in silico pool is the key to evaluation, but the description of the pool generation is too brief. The script of pool generation should be provided.

6. The figure legend of Supplementary Figure 13 is missing.

7. The sentence at line 44 of Page 15 should be rewritten.

**Reviewer 2**

**Were you able to assess all statistics in the manuscript, including the appropriateness of statistical tests used?**

Yes, I was able to assess the statistics.

**Were you able to directly test the methods?**

Yes.

**Comments to author:**

Demultiplexing is an essential step in processing of data where samples from multiple individuals are pooled. In this step, each droplet can be assigned to an individual or considered a doublet. Doublet detection methods try to flag droplets that may contain more than one cell, help with annotation of cells, quality control, and in some cases, understanding the underlying biological process. The authors have performed a benchmarking of both demultiplexing and doublet detection tools, recommend intersectional pipelines using multiple demultiplexing and doublet detection methods for maximum doublet removal, and propose Demuxafy as an integrated tool for demultiplexing and doublet detection in single-cell RNA-seq data. The paper is easy to read and follow. I commend the authors for the extensive work that benchmarking studies involve.

In its current form, Demuxafy seems most helpful for the special case when doublets are a predominant feature of the dataset. The biggest challenge is in interpretation of the benchmarking results, and how to readily adapt and apply them in practice for every single dataset.

Major comments/suggestions:

(1) The authors have chosen to classify (intersectional) methods based on number of droplets captured, and the number of individuals multiplexed. Why were these parameters chosen and why do the individual methods not scale with these parameters?

(2) Another method "Chord" is also based on an ensemble method (for only doublet detection): https://www.nature.com/articles/s42003-022-03476-9 How does demuxafy compare?

(3) How does the classic mouse/human combined dataset (where the doublet ground truth is more readily known) perform in the benchmarking?

(4) How much time does it take to run each of the intersectional methods?

(5) What happens when one subsets the dataset and then runs these pipelines on individual subsets and then consolidate results? Subsetting does not happen randomly, rather based on biology. How dependent are these methods on the biology?

(6) The results are not very easily interpretable because of the complex visualization. The colors are not very easy to distinguish the different methods.For e.g., the colors make it hard to conclude "Scds, Solo, ScDblFinder and DoubletDetection are among the top-performing doublet detecting methods"

(7) Fig 2c: it's a bit hard to read the numbers on the Y-axis, can the final intersections be log2 transformed? How many singlets are uniquely captured? Is there anything about the biology of these singlets? There are a few singlets that doubletdecon or doubletfinder don't accurately assign.

(8) Fig 5 in its current form is not very helpful and reading the decision flow (Fig 5) requires the reader to memorize colors. Can this be better visualized just as a flow chart?

(9) Perhaps the most useful contribution of this paper is the Demuxafy documentation and platform. However, in its current shape it is less user friendly as users must read the paper and make all decisions themselves. Can you provide a form where the user inputs the expected cell numbers, number of individuals and then you provide the recommended intersectional pipeline? Or even have the option to run as many methods as they choose at once and have all the results combined? A tutorial page would make Demuxafy more user friendly.

Other:

(1) Was empty drops incorporated in the Cellranger pipeline (maybe not since the version seems quite old)? If not, then the latest version of Cellranger should be used which uses Emptydroplet filtering to check how this impacts any demultiplexing or doublet detection.

(2) For DoubletDecon and DoubletFinder, the data were preprocessed per developer recommendations. However, results must also be assessed after preprocessing including parameters for clustering should be consistent.

(3) How integrated is Demuxafy to current pipelines in Seurat and Scanpy? How big of a problem are doublets (are they really a bug or feature?) to ask analysts to pivot between Demuxafy and their favorite pipeline?

Minor:

(1) It's a bit unclear why the methods for published datasets are described again.

(2) Re: computational complexity, it is worthwhile to mention that scSplit takes on the order of days and Solo on the order of hours in the text.

**Authors Response**

**Point-by-point responses to the reviewers’ comments:**

Reviewer #1

In the manuscript, the authors compare the performance of doublet and singlet annotation of current demultiplexing and doublet detection methods, focusing on large scRNA-Seq datasets generated from pooled cells from multiple individuals. By benchmarking the performance of the methods in two real datasets and simulated datasets in sillico, the authors conclude that it is unreliable to apply only one type of method on this type of data. Therefore, the authors integrate selected tools into a framework Demuxafy to improve the droplet assignment by intersectional approach. The authors also provide a brief guidance of the tool combinations when facing different experimental design.

While multiple tools are available for genetic demultiplexing, this is the first work seen benchmarking these tools. Plus, the authors raise a good point that demultiplexing methods and doublet detection methods are complementary in droplet assignment when genetically mixed cells are pooled and sequenced. However, an alternative strategy of integrating two types of methods should be considered. Due to the nature of design, demultiplexing methods are good at dealing with heterogenic doublets while doublet detection ones are good at detecting heterotypic doublets. Therefore, the modules integrating best performed demultiplexing methods and integrating best performed doublet detection methods can be included in the framework separately. When dealing with large scRNA-Seq datasets from genetically mixed cells, the sequential application of two modules can be employed to achieve better result. This will also make the framework more flexible. This strategy should be tested or at least discussed.

*Reply: Thank you for the thoughtful review of our manuscript. We appreciate your feedback and are grateful that you view the topic as informative and timely.*

Specific points:

1. The authors ignore the previous comprehensive benchmarking report of doublet detection methods (Cell Syst. 2021 Feb 17;12(2):176-194.e6), which shows that DoubletFinder has the overall best performance. However, this method is not integrated in the framework as shown in Figure 5. The authors should justify the reason.

*Reply: Thank you for raising this point. In the first submission, had originally only considered demultiplexing and doublet detecting methods that demonstrated the best performance as single methods for integration with other methods during the intersectional analysis. DoubletFinder was not among those methods. However, recognising the work presented in Xi and Li (2021), we have expanded our analysis to include all methods. Our results demonstrate that DoubletFinder performs well under specific conditions but may not be as consistent as other methods.*

2. Similarly, the selection of the four demultiplexing tools into the framework is not justified.

*Reply: We apologise for the omission here. We have now expanded the analysis to include all possible software comparisons (>60k) and provide the updated results in the ‘Performances Vary Between Demultiplexing and Doublet Detecting Method and Across the Number of Multiplexed Individuals’ section.*

3. In Figure 2a-b, it is unclear what does the agreement mean and how the score is generated. It is specifically obscure that the authors state the agreement is calculated in different ways when demultiplexing methods are compared vs. when doublet detection methods are compared. If so, it is inappropriate to have them in the same heatmap. It shows that DoubletDecon and DoubletFinder have clear disagreements with other methods in both datasets. Does it mean one or both methods are not reliable?

*Reply: We thank Reviewer #1 for pointing out this missing information. The score used as the comparison metric is the percentage of consistently annotated droplets (between two methods). We have now added this information to clarify what we are showing in the ‘Demultiplexing and Doublet* *Detecting Methods Perform Similarly for Heterogeneous and Homogeneous Cell Types’ section. The information is now displayed in* ***Supplementary Figure 1****.*

4. In Supplementary Figure 1, the authors show the correlation of every two methods. It demonstrates a wide range of correlation in doublet detection methods. The detailed correlation results should be included in a table.

*Reply: We thank Reviewer #1 for this helpful suggestion. We have now included the percent agreement metrics in* ***Supplementary Tables 2-3****.*

5. The in silico pool is the key to evaluation, but the description of the pool generation is too brief. The script of pool generation should be provided.

*Reply: We thank Reviewer #1 for flagging this, and we apologise for briefly explaining the in silico pool methods. We have extended the simulation methods; all scripts are provided on Github and Zenodo.*

6. The figure legend of Supplementary Figure 13 is missing.

*Reply: We apologize for the accidental omission of one of the Supplementary Figure legend. We have now ensured that all the Supplementary Figure legends are present.*

7. The sentence at line 44 of Page 15 should be rewritten.

*Reply: Unfortunately, we were unable to identify the exact sentence Reviewer #1 was referring to since we did not have the same numbering system on our manuscript version as the reviewer was referring to. Therefore, we are guessing that the sentence that the reviewer was referring to was this sentence: “We regarded as multiple experiment designs and provided recommendations on two different levels of filtering doublets: 1) a balanced approach that attempts to remove true doublets while not removing too many true singlets (assessed with the MCC) and 2) an approach that removes as many doublets as possible even if some droplets that are true singlets are classified as doublets (assessed with the positive predictive value [PPV]).” We hope that this has addressed the reviewer’s concern.*

Reviewer #2

Demultiplexing is an essential step in processing of data where samples from multiple individuals are pooled. In this step, each droplet can be assigned to an individual or considered a doublet. Doublet detection methods try to flag droplets that may contain more than one cell, help with annotation of cells, quality control, and in some cases, understanding the underlying biological process. The authors have performed a benchmarking of both demultiplexing and doublet detection tools, recommend intersectional pipelines using multiple demultiplexing and doublet detection methods for maximum doublet removal, and propose Demuxafy as an integrated tool for demultiplexing and doublet detection in single-cell RNA-seq data. The paper is easy to read and follow. I commend the authors for the extensive work that benchmarking studies involve.

In its current form, Demuxafy seems most helpful for the special case when doublets are a predominant feature of the dataset. The biggest challenge is in interpretation of the benchmarking results, and how to readily adapt and apply them in practice for every single dataset.

*Reply: Thank you for your time reviewing our manuscript and insightful feedback. Based on your comments, we have heavily revised the manuscript, focusing on the interpretation of the benchmarking results, the flow of the narrative, and the simplification of figures where possible. We believe these changes improve the manuscript's accessibility and utility for non-expert and expert readers.*

*Once again, we sincerely appreciate your time and thoughtful feedback, and we hope our work will contribute to the genetics community.*

Major comments/suggestions:

(1) The authors have chosen to classify (intersectional) methods based on number of droplets captured, and the number of individuals multiplexed. Why were these parameters chosen and why do the individual methods not scale with these parameters?

*Reply: Thank you for this question. We allowed the data to drive the grouping of the results. Even though the same combinations of demultiplexing and doublet detecting methods were applied to each of the simulated datasets, the best-performing combination of methods needed to be more consistent for all experimental designs ranging in the number of pooled donors and droplets captured, this is because the number of doublets is a function of the size of the pool and the number of donors present. We observed that some combinations of methods performed well only until a certain number of donors were pooled or a certain number of droplets were captured. Therefore, we opted to provide nuanced suggestions based on the performance of the combined methods for each experimental condition. Our view is that a user will need to decide on the best group of methods based on their experimental design* *– and through this, optimise the ability to demultiplex and identify doublets. We have revised the text to improve our understanding of using Demuxify under different scenarios.*

(2) Another method "Chord" is also based on an ensemble method (for only doublet detection): [https://www.nature.com/articles/s42003-022-03476-9](https://protect-au.mimecast.com/s/xKsPCr81vOuAvZD2FmXIeB?domain=nature.com) How does demuxafy compare?

*Reply: We thank the reviewer for this recommendation. We have now compared Chord to Demuxafy and find that Demuxafy performs similarly to Chord for smaller pools and outperforms Chord in larger pools. We observed that this is because Demuxafy reduces the number of droplets falsely annotated as singlets or doublets. We have now included these results in* ***Figure S14*** *and commentary in the text.*

(3) How does the classic mouse/human combined dataset (where the doublet ground truth is more readily known) perform in the benchmarking?

*Reply: We thank Reviewer #2 for this recommendation. We feel that a human/mouse combination experiment would not be reflective of single species demultiplexing because the two species have different genomes and genetic variants. Even though we could generate a genetic map that contains both mouse and human SNPs, the variants used for human cells would be completely different from the variants used for mouse cells and, therefore, doesn’t test the same challenges that are observed with single-species demultiplexing. Therefore, we do not feel the human/mouse experiment results would add value. However, in Xi and Li’s (2021, Cell Systems) comparison of doublet detection methods, this the mixed mouse / human experiment was used to evaluate doublet detection methods.*

(4) How much time does it take to run each of the intersectional methods?

*Reply: The time will depend on the pool size and computational infrastructure. We have provided the range of running times for pools of ~20k for each software in* ***Figure S3*** *so readers can estimate the expected execution times depending on their system's experiment.*

(5) What happens when one subsets the dataset and then runs these pipelines on individual subsets and then consolidate results? Subsetting does not happen randomly, rather based on biology. How dependent are these methods on the biology?

*Reply: Good question. Since the transcription-based doublet detecting methods depend on the random combination of different cell types in the pool to inform the profiles of doublets in the dataset, it would be a disadvantage to subset the cells based on cell type as the reviewer suggests. The demultiplexing methods would be less impacted by biological subsetting of the data since it is based on genetic information which will be consistent in different cell types. We have mentioned this in the discussion.*

(6) The results are not very easily interpretable because of the complex visualization. The colors are not very easy to distinguish the different methods.For e.g., the colors make it hard to conclude "Scds, Solo, ScDblFinder and DoubletDetection are among the top-performing doublet detecting methods"

*Reply: We thank Reviewer #2 for pointing out the issue with our color pallet as we strive to make the results as clear and interpretable as possible. We have now updated the color scheme to make it easier to identify the different methods on each figure. We hope that the new colors we have selected are easier to differentiate.*

(7) Fig 2c: it's a bit hard to read the numbers on the Y-axis, can the final intersections be log2 transformed? How many singlets are uniquely captured? Is there anything about the biology of these singlets? There are a few singlets that doubletdecon or doubletfinder don't accurately assign.

*Reply: We thank Reviewer #2 for raising these points. We agree that the information in the original* ***Figure 2c*** *could have been better communicated in a different format, so we have now replaced it with* ***Figures 2a-d****. Since it was not possible to understand the factors contributing to differences in singlet/doublet assignment for each method, we opted to interrogate this question following the simulation. This allowed us to properly assess how different cell types and doublet combinations were potentially biased by specific methods. This information is included in* ***Supplementary Figures 6-7****.*

(8) Fig 5 in its current form is not very helpful and reading the decision flow (Fig 5) requires the reader to memorize colors. Can this be better visualized just as a flow chart?

*Reply: We thank Reviewer #2 for their comment on* ***Figure 5*** *and have now updated the figure to simplify the visualization.*

(9) Perhaps the most useful contribution of this paper is the Demuxafy documentation and platform. However, in its current shape it is less user friendly as users must read the paper and make all decisions themselves. Can you provide a form where the user inputs the expected cell numbers, number of individuals and then you provide the recommended intersectional pipeline? Or even have the option to run as many methods as they choose at once and have all the results combined? A tutorial page would make Demuxafy more user friendly.

*Reply: We appreciate the suggestions from Reviewer #2 to enhance the Demuxafy documentation. We have now extended our expected doublet estimator to include recommended software based on experimental design. In addition, we have simplified the documentation and added a tutorial in v2.0.1.*

Other:
(1) Was empty drops incorporated in the Cellranger pipeline (maybe not since the version seems quite old)? If not, then the latest version of Cellranger should be used which uses Emptydroplet filtering to check how this impacts any demultiplexing or doublet detection.

*Reply: As the reviewer points out, the Cellranger version used is not the newest since it was the version used to process the data originally (v2.2.0) and did not use EmptyDrops which was introduced in Cellranger v3.0.0. We have included results from Cellbender that demonstrate that the number of empty droplets included in this analysis is extremely low (~0.03% of the PBMC droplets and ~0.35% of the fibroblast droplets) indicating that empty droplets likely would not impact the demultiplexing or doublet detecting results.*

(2) For DoubletDecon and DoubletFinder, the data were preprocessed per developer recommendations. However, results must also be assessed after preprocessing including parameters for clustering should be consistent.

*Reply: As pointed out by Reviewer #2, DoubletDecon and DoubletFinder are dependent on a number of parameters that the user must select. To address this issue, we executed DoubletDecon with multiple rhop values to identify the most appropriate rhop for each pool and used the pK vs BC_MVN_ plotting suggested by DoubletFinder to select the most appropriate pK value for Doublet Finder. We have extended the text in the Methods sections to more clearly describe this.*

(3) How integrated is Demuxafy to current pipelines in Seurat and Scanpy? How big of a problem are doublets (are they really a bug or feature?) to ask analysts to pivot between Demuxafy and their favorite pipeline?

*Reply: We thank Reviewer #2 for this query. Demultiplexing and doublet detection should be executed before any quality control analysis. Demuxafy is very easy to integrate with popular single-cell tools such as Seurat, Scanpy and Loom and we have provided users with pseudocode to add the results from Demuxafy directly to objects for any of these packages on the Demuxafy website:* [*https://demultiplexing-doublet-detecting-docs.readthedocs.io/en/latest/NextSteps.html*](https://demultiplexing-doublet-detecting-docs.readthedocs.io/en/latest/NextSteps.html) *and have also included this in the Tutorial. In addition, for those users who would prefer to run any doublet detecting tools directly in R or Python, we have provided the code on the website so they can build it directly into their scripts with either Seurat or Scanpy providing flexibility based on user comfort.*

Minor: 
(1) It's a bit unclear why the methods for published datasets are described again.

*Reply: We provide just very brief descriptions of each dataset so that the readers can easily find the information without searching for another manuscript. This is also helpful for research consistency as well.*

(2) Re: computational complexity, it is worthwhile to mention that scSplit takes on the order of days and Solo on the order of hours in the text.

*Reply: We thank the reviewer for this recommendation and have updated the text accordingly.*

**Second round of review**

**Reviewer 1**

The manuscript is significantly improved. All my concerns are addressed.

**Editorial board member**

The authors addressed all the concerns of the referee 2, apart from (3) and (5), but there I am inclined to agree with the authors' responses and that doing so would not add significantly to the manuscript and need not be further addressed.
